# NMR as a Tool to Investigate the Processes of Mitochondrial and Cytosolic Iron-Sulfur Cluster Biosynthesis

**DOI:** 10.3390/molecules23092213

**Published:** 2018-08-31

**Authors:** Kai Cai, John L. Markley

**Affiliations:** Biochemistry Department, University of Wisconsin-Madison, Madison, WI 53706, USA; kcai@wisc.edu

**Keywords:** NMR, iron-sulfur cluster biogenesis, mitochondria, ISC, CIA

## Abstract

Iron-sulfur (Fe-S) clusters, the ubiquitous protein cofactors found in all kingdoms of life, perform a myriad of functions including nitrogen fixation, ribosome assembly, DNA repair, mitochondrial respiration, and metabolite catabolism. The biogenesis of Fe-S clusters is a multi-step process that involves the participation of many protein partners. Recent biophysical studies, involving X-ray crystallography, nuclear magnetic resonance (NMR) spectroscopy, mass spectrometry (MS), and small angle X-ray scattering (SAXS), have greatly improved our understanding of these steps. In this review, after describing the biological importance of iron sulfur proteins, we focus on the contributions of NMR spectroscopy has made to our understanding of the structures, dynamics, and interactions of proteins involved in the biosynthesis of Fe-S cluster proteins.

## 1. Introduction

Metal ions are essential to life and, to function, almost half of all enzymes must associate with one or more particular metal ions [1,2]. Iron-sulfur (Fe-S) clusters are among the most ancient yet ubiquitous protein cofactors present in all kingdoms of life. The most common types of Fe-S clusters are [2Fe-2S], [3Fe-4S], and [4Fe-4S], while more complex cluster forms, such as the [8Fe-7S] cluster (P-cluster) and iron-molybdenum cofactor (FeMoco) of nitrogenase, have also been reported [3,4,5]. The physical and chemical properties of Fe-S clusters enable them to play important roles in a variety of biological pathways, including respiration, DNA repair and replication, gene regulation, central metabolism, and RNA modification [6,7]. Fe-S clusters display large span of redox potentials and are often used for electron transfer in cellular processes [8,9,10,11]. Fe-S clusters also play important roles in a variety of non-redox cellular functions [12]. The instability of their clusters allows Fe-S proteins to function as biological sensors in the regulation of cellular iron homeostasis: examples include the bacterial transcription factors FNR and IscR, and cytosolic aconitase (IRP1) in the IRP1-IRE system [13,14,15]. The [4Fe-4S] cluster on mitochondrial aconitase, on the other hand, functions as a Lewis-acid catalyst in the mitochondrial tricarboxylic acid (TCA) cycle [16]. Fe-S clusters are required for the function of several nucleic acid processing enzymes, including glycosylases, primases, helicases, nucleases, transcription factors, RNA polymerases, and RNA methyltransferases, although the functions of their Fe-S clusters are poorly understood [17,18]. Loss of Fe-S proteins has been linked to genome instability [19]. It has shown that the Fe-S cluster in primase serves as a redox switch for DNA binding [18]. The eukaryotic replicative DNA polymerases require a Fe-S cluster for replisome assembly and stability [20]. Fe-S clusters are also essential for DNA repair helicases XPD and FancJ [21]. Fe-S proteins are involved in the metabolism of nucleotides, such as dihydropyrimidine and flavin adenine dinucleotide [22]. The Fe-S protein ABCE1, which plays important roles in ribosome biogenesis and maturation, translation initiation and termination, and ribosome recycling, has been found to be essential to cell viability [23,24]. S-adenosyl-l-methionine (SAM)-dependent enzymes, such as lipoic acid synthase and biotin synthase, require Fe-S clusters for their functions [25,26,27]. Recently, a Fe-S protein, *Drosophlia* MagR (the same as ISCA1), has been proposed to be the magnetosensing protein in biological systems [28].

Although Fe-S clusters can be synthesized in vitro without enzymatic assistance, the biogenesis of Fe-S cluster in cellular environments is a highly regulated, multistep pathway that involves many proteins. Our best understanding of the Fe-S cluster biosynthetic mechanism stems from studies in bacteria, in which three machineries have been described: the NIF (nitrogen fixation) system, the ISC (iron-sulfur cluster) system, and the SUF (sulfur utilization factor) system [4,5,7,29]. Of these, the NIF system is specialized for nitrogen fixation, and the SUF system is utilized primarily under oxidative stress or iron starvation conditions. This leaves the ISC system as the “house-keeping” machinery for bacterial Fe-S cluster biogenesis. The ISC machinery, encoded by the *iscRSUA* operon, contains the following components: IscR (transcription factor that regulates the expression of the iscRSUA operon in response to the iron-sulfur cluster content of the cell), IscS (cysteine desulfurase), IscU (scaffold protein for de novo Fe-S cluster assembly), IscA (A-type Fe-S cluster carrier protein), Fdx (electron donor), IscX (iron-binding protein and putative iron donor), HscA (specialized Hsp70 chaperone), HscB (Hsp40 J-type co-chaperone) [5,7,30]. Another protein, which is not encoded by the *iscRSUA* operon but is important to the bacterial ISC machinery, is the iron-binding protein CyaY, the bacterial homolog of frataxin (FXN). CyaY has been shown to be a negative regulator of Fe-S cluster biosynthesis in *Escherichia coli* (*E. coli*) [31,32]. These components form an intricate protein–protein interaction network that facilitates Fe-S cluster assembly and transfer [33].

**Figure 1:**
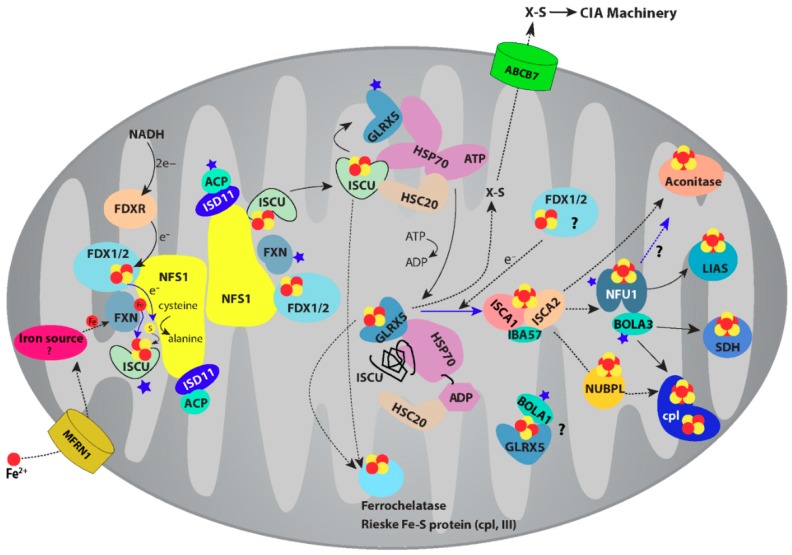
Schematic representation of the current model of human mitochondrial Fe-S cluster biogenesis. Iron enters the mitochondrion via the iron transporter MFRN1. The cysteine desulfurase NFS1 (N) exists in a dimeric form and mobilizes sulfur from l-cysteine for Fe-S cluster assembly. The accessory protein ISD11 (I) and acyl carrier protein ACP (A) are required for the function and stability of NFS1. The scaffold protein ISCU (U), frataxin FXN (F), and ferredoxin FDX2 (X) all bind to the surface of NFS1 to form the (NIAUXF)_2_ complex. FXN is the proximal iron donor, which receives Fe^2+^ from an unidentified iron source. FXN regulates the cysteine desulfurase activation of NFS1 and iron entry to ISCU. One electron to reduce S^0^ to S^2−^ is provided by an electron transport chain consisting of NAD(P)H, ferredoxin reductase (FDXR) and ferredoxin (FDX1/2). The other electron is likely provided by the oxidation of Fe^2+^ bound to FXN. The assembled [2Fe-2S] cluster is transferred from ISCU to a monothiol glutaredoxin GLRX5 assisted by the dedicated chaperone–cochaperone (HSP70-HSC20) system. Subsequently, Fe-S clusters are either directly inserted into mitochondrial [2Fe-2S] proteins (e.g., Rieske protein), used for synthesis of a sulfur-containing species (X-S) for cytosolic Fe-S cluster assembly (CIA machinery). Assembly of [4Fe-4S] clusters involves ISCA1, ISCA2, and IBA57. An electron donor, such as ferredoxin, may be required to convert two [2Fe-2S] clusters to a [4Fe-4S] cluster. Late-acting factors, such as NFU1 and NUBPL, are required for the maturation of a subset of [4Fe-4S] clusters, such as LIAS and SDH. The mitochondrion export of the unidentified sulfur-containing compound (X-S) is facilitated by an ABC transporter ABCB7. In the cytoplasm, the cytosolic Fe-S cluster assembly machinery (CIA) inserts Fe-S clusters into cytosolic and nuclear Fe-S proteins. The blue stars denote the proteins (or the protein homologs) for which the structures were obtained by NMR, and blue arrows denote the processes that were clarified by NMR.

In nearly all eukaryotes, mitochondria are the major compartments for Fe-S cluster biogenesis. Eukaryotic mitochondria possess an ISC machinery that was inherited from bacteria through an endosymbiotic process and is highly conserved from yeast to man [34]. The ISC machinery in human mitochondria consists of at least 19 known proteins and can be divided into several steps (Figure 1 and Table 1). The first step is *de novo* assembly of a nascent [2Fe-2S] cluster on the scaffold protein ISCU through coordinated reactions involving a set of essential ISC proteins: the cysteine desulfurase (NFS1), an accessory protein (ISD11), the mitochondrial acyl carrier protein (ACP), ferredoxin (FDX1/2), and frataxin (FXN) [24,35,36,37]. The second step has been proposed to be release of nascent [2Fe-2S] cluster from the scaffold protein to the mitochondrial monothiol glutaredoxin (GLRX5) facilitated by the mitochondrial chaperone/cochaperone system [38,39]. The [2Fe-2S] clusters can be subsequently transferred to target proteins, trafficked to late acting protein complexes to form [4Fe-4S] clusters, or exported from mitochondria as a sulfur-containing species used in the cytosolic iron-sulfur assembly (CIA) machinery [40,41,42]. [4Fe-4S] cluster synthesis is accomplished by a set of proteins, including ISCA1, ISCA2, and IBA57 [43,44,45]. Once synthesized by ISCA proteins, [4Fe-4S] clusters are inserted into [4Fe-4S] protein targets such as aconitase, respiratory complex I, and lipoic acid synthase. The insertion of [4Fe-4S] cluster to target proteins involves other ISC proteins such as NFU1, BOLA3, and NUBPL. The exact functions of these proteins are not yet clearly defined, but they have been proposed to be the intermediate [4Fe-4S] carriers and late acting factors that are essential for the maturation of specific [4Fe-4S] proteins [46,47,48]. 

Defects in protein components of the mitochondrial ISC machinery are associated with numerous diseases, including Friedreich ataxia (defects in frataxin), myopathy (defects in ISCU or FDX2), and multiple mitochondrial dysfunction syndromes (defects in NFU1, BOLA3, ISCA2, and IBA57) [24,49,50,51]. Extensive investigations over the past two decades have identified many new components and established key steps in the ISC machinery (Table 1). A growing number of diseases associated with ISC defects are being discovered through clinical, genetic, and biochemical studies. 

Solution NMR spectroscopy has been employed frequently in elucidating protein structures, dynamic properties, and protein–protein interaction networks involved in Fe-S cluster assembly and transfer. NMR is a powerful method for studying paramagnetic proteins such as rubredoxins, HiPIPs, and ferredoxins [52]. The first solution NMR structure of a paramagnetic protein was that of *E. halophila* HiPIP I [53]. Many NMR experiments developed to investigate paramagnetic proteins have been successfully applied to studies of proteins containing Fe-S clusters. Experiments tailored for paramagnetic systems include those designed for the detection of NMR hyperfine shifts [54,55], the measurement of longitudinal (*R*_1_) and transverse (*R*_2_) relaxation [56], the direct detection of ^13^C signals [57,58], the collection of 2D HSQC and IR-^15^N-HSQC-AP data, where HSQC stands for heteronuclear single-quantum correlation, IR stands for inversion recover, and AP stands for antiphase [59,60]. NMR is also very effective in characterizing weak or transient protein–protein interactions [61], which are prevalent in the myriad of protein–protein interactions leading to Fe-S cluster synthesis and transfer [59,62,63]. 

In this review, we discuss recent advances in the field of Fe-S cluster biogenesis and contributions NMR has made toward our understanding of these processes. We focus on the human mitochondrial ISC and cytosolic CIA machineries. However, because much of our understanding of these human machineries stems from studies of the bacterial and yeast ISC and CIA machineries, these systems are briefly mentioned. 

## 2. Fe-S Cluster Assembly on A Scaffold Protein as Studied by NMR Spectroscopy

**Figure 2:**
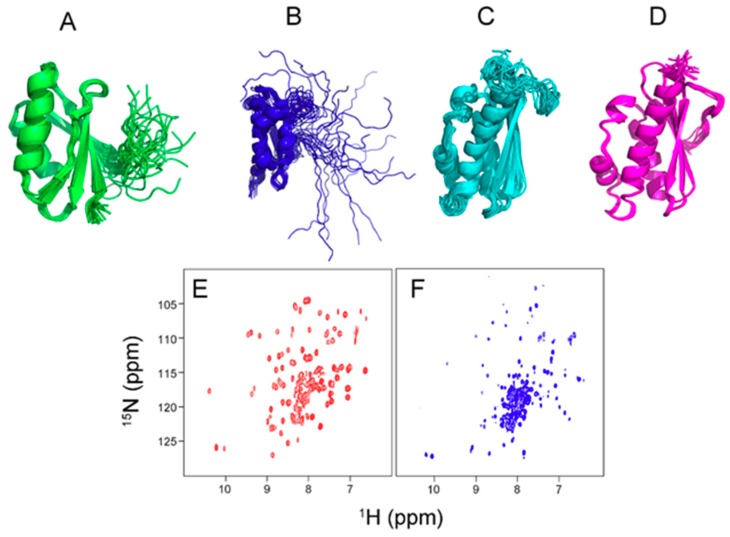
NMR studies of ISCU proteins. Solution NMR structures of: (**A**) *E. coli* apo-IscU(D39A), a variant that is fully in the structured state (PDB: 2KQK); (**B**) the structured-form of wild-type *E. coli* apo-IscU (PDB: 2L4X); (**C**) zinc-bound *Haemophilus Influenzae* IscU (PDB: 1R9P); and (**D**) zinc-bound *Mus musculus* ISCU (PDB: 1WFZ) [87,88]. Zinc-binding stabilizes the structured state. ^1^H-^15^N HSQC spectra of: (**E**) *E. coli* apo-IscU; and (**F**) human apo-ISCU at pH 7.5 and 25 °C. These spectra contain signals from both the structured and disordered states of the protein and show that human ISCU is more disordered than *E. coli* IscU. See Refs. [84,85] for details.

The first step of mitochondrial ISC machinery is the *de novo* assembly of a [2Fe-2S] cluster on the scaffold protein ISCU through coordinated reactions involving a set of essential ISC proteins (NFS1, ISD11, ACP, ferredoxin, and FXN). ISCU is a highly conserved protein, which contains three cysteine residues whose sulfhydryl groups serve to ligate the Fe-S cluster; the fourth ligand appears to be either a histidine or aspartate side chain. Owing to the conformational flexibility of ISCU, NMR has become the go-to approach to study the structures and dynamics of ISCU. NMR studies from our laboratory have shown that apo-ISCU and its *E. coli* homolog (IscU) are metamorphic proteins that populate two interconverting conformations: A structured state (S-state) and a dynamically disordered state (D-state) [83,84,85,86]. Several structures have been determined by NMR of the structured state of the protein [87,88] (Figure 2A–D). One structure is of the single-site *E. coli* variant IscU(D39A) (Figure 2A). Another is the structure is of the S-state of wild-type IscU determined in the presence of the D-state (Figure 2B). Two others are of zinc-binding complexes, which stabilize the S-state (Figure 2C,D). In these Zn-ISCU complexes, Zn ion binds to same pocket as the Fe-S cluster with *K*_d_ estimated to be around 10^−13^ M [89]. Recent in vitro studies have suggested a potential physiological function of Zn^2+^-ISCU. Zn has been shown to inhibit in vitro Fe-S cluster assembly [85]. It has been shown that Zn can modulate the cysteine desulfurase function in an ISCU-dependent manner [90,91]. A possible explanation is that Zn occupies Fe-S cluster binding site of ISCU, thus preventing sulfur transfer and Fe-S formation. Recent X-ray structures of human cysteine desulfurase-ISCU complex showed that the disordered active site cysteine-containing-loop becomes structured at the presence of Zn [92]. These in vitro studies suggest a possible connection between Zn metabolism and Fe-S cluster biogenesis. Zn has been shown to inhibit the mitochondrial TCA cycle and electron transport chain [93,94], both of which depend on mitochondrial ISC machinery. Future in vivo studies are needed to verify and further establish this connection. 

Despite their high (77%) sequence identity, the S:D ratios are quite different for *E. coli* IscU (80:20) (Figure 2E) and human ISCU (25:75) [84,85] (Figure 2F). The lifetimes of the S- and D-states are each on the order of one second, and their interconversion can be measured by using ZZ-exchange NMR experiments [85]. The S- and D-states of *E. coli* IscU were found to differ by the configurations of the N13-P14 and P100-P101 peptide bonds, which are *trans* in the S-state and *cis* in the D-state [95]. The reason the scaffold protein has evolved to populate two conformations remains a mystery. However, clues come from evidence for preferential binding of different proteins to the S- and D-states. Our NMR studies have shown that the J-type co-chaperone (*E. coli* HscB, human HSC20) binds preferentially to the S-state; the Hsp70-type chaperone protein (*E. coli* HscA, human HSP70) binds preferentially to the D-state [84,96]; and the cysteine desulfurase *(E. coli* IscS, human NFS1) binds both states with preference for the D-state [84,85]. Others have questioned whether IscS binds to the D-state, and have presented NMR evidence for only the S-state in the complex [97]. We are committed to reinvestigating this question by ^19^F-NMR studies of [^19^F-Trp^76^]-IscU. 

The sulfur atoms used in Fe-S cluster biosynthesis are generated from the enzymatic conversion of l-cysteine to l-alanine by cysteine desulfurase. Mitochondrial cysteine desulfurase (NFS1) is a pyridoxal phosphate (PLP)-dependent enzyme that mobilizes sulfur from l-cysteine to form a persulfide intermediate on the conserved active-site cysteine of NFS1 [98]. Unlike its bacterial homolog IscS, the full function and stability of NFS1 requires other two accessory proteins, namely ISD11 and ACP. A recent study has shown that the activity of NFS1 is also regulated by phosphorylation [99]. ISD11 (or LYRM4) is a member of the LYRM (Leu-Tyr-Arg Motif) family proteins and is important for both mitochondrial Fe-S cluster biogenesis and iron homeostasis [100,101,102]. LYRM family proteins are small, basic proteins that carry a conserved Leu-Tyr-Arg sequence close to their N-terminus. The human genome contains at least ten LYRM proteins that localize predominantly to mitochondria [103,104]. ACP is a small, acidic protein known to function in mitochondrial fatty acid biosynthesis (FASII) through reactions involving its 4′-phosphopantethiene (4′-PPT) cofactor, which is conjugated to a conserved serine residue [105,106]. ACPs are universal and highly conserved proteins that are nature’s way of transporting hydrocarbon chains in vivo [107]. ACPs are dynamic proteins. Multiple structures of acyl carrier proteins in different forms (apo-, holo- or acylated) from different organisms have been obtained via solution NMR [108,109,110,111,112]. The dynamic acyl chain-flipping mechanism of ACPs and the transient interactions between ACPs and other proteins have also been studied by solution NMR [113,114,115]. To date, no structure of ISD11 on its own has been determined. It may be that ISD11 becomes stably structured only when it binds to ACP [116]. A possible mechanism is that ACP stabilizes the structure of ISD11 by extending its acyl chain into the α-helical bundle of ISD11. Our NMR data suggest that ISD11 is intrinsically disordered by itself and becomes structured upon binding ACP [117]. Earlier, Yan et al. claimed to have successfully isolated and purified ISD11, and their published ^1^H-^15^N HSQC spectrum of the protein indicated that it was well-structured [118]. However, our examination of this ^1^H-^15^N HSQC spectrum revealed that it closely matches that of *E. coli* Acp rather than ISD11 [119]. Thus, we suggest that the protein purified by the authors of this study was *E. coli* Acp, which is known to associate with ISD11 [103], and was attributed mistakenly to ISD11. The authors also reported that their purified protein interacts with *E. coli* IscS [118]. Because an interaction between Acp and IscS had been identified previously by tandem affinity purification [120], this finding is also consistent with the isolated protein being Acp. The Acp-IscS interaction is likely to be non-specific as none of the known IscS structures has Acp as its accessory protein [121,122,123], and our LC-MS/MS and amino acid quantification experiments failed to identify Acp from purified IscS [117].

**Figure 3:**
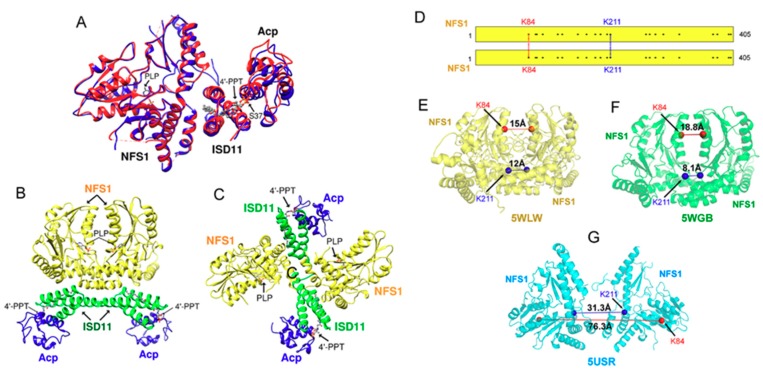
Investigation of structures of (NIA)_2_ and (NIAU)_2_ by X-ray crystallography and cross-linking as analyzed by mass spectrometry (XL-MS). (**A**) Overlay of one-half (NIA)_1_ of each of two independently-determined X-ray structures of the human cysteine desulfurase complex (NIA)_2_: red, PDB entry 5WGB [92]; blue, PDB entry 5USR [125]; (**B**) X-ray structure of the full human cysteine desulfurase complex (NIA)_2_ by Boniecki et al. (PDB: 5WGB) showing its “closed” conformation [92]. (**C**) X-ray structure of full human cysteine desulfurase complex (NIA)_2_ by Cory et al. (2017) (PDB: 5USR) showing its “open” conformation [125]; (**D**) Experimental inter-subunit NFS1-NFS1 crosslinks in (NIAU)_2_ as determined by XL-MS [129]. The maximum C^α^-C^α^ distance expected for such crosslinks is 27.4 Å. This expected distance is compatible with C^α^-C^α^ distances in the structures (**E**) of (NIAU)_2_ (PDB: 5WLW) and (**F**) of (NIA)_2_ (PDB: 5WGB) by Boniecki et al. [92] but not with C^α^-C^α^ distances in the structure (**G**) of (NIA)_2_ (PDB: 5USR) by Cory et al. (2017) [125]. The positions of the crosslinked lysine residues are indicated by spheres, and, for clarity, only the two NFS1 subunits are shown. Adapted from Ref. [129] with permission.

The role of ACP in mitochondrial Fe-S cluster biogenesis has recently come to light. In yeast, Acp1 was found to be an essential component of cysteine desulfurase complex [124]. We demonstrated that *E. coli* Acp substitutes for human mitochondrial ACP in the cysteine desulfurase complex produced by co-expressing human NFS1 and ISD11 in *E. coli* cells and determined its stoichiometry to be [NFS1]_2_:[ISD11]_2_:[Acp]_2_ [117], henceforth abbreviated as (NIA)_2_. Two independent crystal structures of (NIA)_2_ were subsequently determined, each of which confirmed this stoichiometry [92,125]. The structures of NFS1, ISD11, and Acp subunits of the (NIA)_2_ complex are very similar in both X-ray structures. In both structures, the conserved “LYR” motif of ISD11 interacts with Acp, and the acylated 4′-PPT cofactor of Acp is flipped-out and extends into the α-helical bundle of ISD11; this configuration closely resembles those of the LYRM-ACP complexes found in structures determined by cryo-EM of mitochondrial respiratory complex I [126,127] and mitochondrial ribosome [128]. However, the quaternary architectures of the two (NIA)_2_ structures are strikingly different. In the (NIA)_2_ structure by Boniecki et al. (PDB: 5WGB), the two NFS1 subunits form a “closed” dimer conformation similar to that of *E. coli* IscS [92,121,122,123]. By contrast, the structure determined by Cory et al. (PDB: 5USR) adopts a unique “open” conformation in which the two NFS1 units have little contact with each other and the substrate-binding site of NFS1 is exposed [125] (Figure 3A–C). Our chemical crosslinking coupled with mass spectrometry (XL-MS) and small angle X-ray scattering (SAXS) data for (NIAU)_2_ are consistent with the “closed” form (Figure 3D–F). We are currently exploring the possibility of structural heterogeneity of (NIA)_2_ in solution by ^19^F-NMR studies of (NIA)_2_ prepared from NFS1 containing ^19^F-labeled Trp. NFS1 contains three Trp residues. Preliminary results show the presence of more than three ^19^F-NMR peaks in spectra of the complex, suggesting potential structural heterogeneity [129]. 

For subsequent Fe-S cluster formation, S^0^ released from l-cysteine by cysteine desulfurase (NIA)_2_ needs to be reduced to sulfide (S^2−^) by the addition of two electrons. The first electron is delivered by ferredoxin (FDX, Yah1 in yeast), and the second is thought to be provided through the oxidation of Fe(II) to Fe(III). FDX is reduced by FDXR (Arh1 in yeast), which utilizes NADPH as its electron source [130,131,132,133]. Human mitochondria possess two ferredoxins, namely FDX1 and FDX2. 

**Figure 4:**
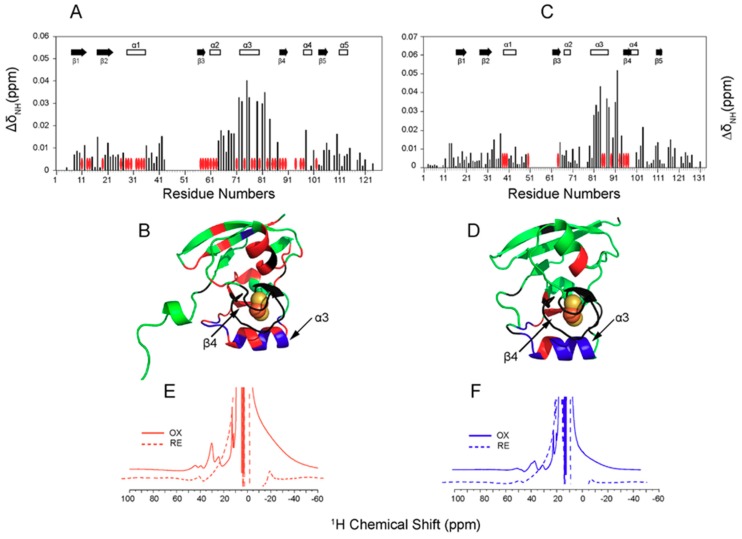
NMR studies of human mitochondrial ferredoxins FDX1 and FDX2 and their interactions with (NIA)_2_. (**A**) Chemical shift (CS) perturbation of the ^1^H-^15^N signals (Δδ_NH_) of [U-^15^N]-FDX1 resulting from the interaction with (NIA)_2_. The red ovals denote the residues whose signals were broadened beyond detection; (**B**) CS perturbation results from (**A**) mapped onto the structure of FDX1 (PDB: 3P1M). Color code: green, not significantly affected (Δδ_NH_ < 0.03 ppm); blue, significant chemical shift changes (Δδ_NH_ > 0.03 ppm); red, severe line broadening; black, no assignments; (**C**) Chemical shift (CS) perturbation of the ^1^H-^15^N signals (Δδ_NH_) of [U-^15^N]-FDX2 resulting from the interaction with (NIA)_2_. The red ovals denote the residues whose signals were broadened beyond detection; (**D**) CS perturbation results from (**C**) mapped onto the structure of FDX1 (PDB: 2Y5C). Color code: green, not significantly affected (Δδ_NH_ < 0.03 ppm); blue, significant chemical shift changes (Δδ_NH_ > 0.03 ppm); red, severe line broadening; black, no assignments; (**E**) 1D ^1^H-NMR spectra of oxidized (OX) and reduced (RE) FDX1 showing hyperfine shifted signals in the 10 ppm to 50 ppm and −10 ppm to −25 ppm regions. (**F**) 1D ^1^H-NMR spectra of oxidized (OX) and reduced (RE) FDX2 showing hyperfine shifted signals in the from 10 ppm to 50 ppm and from −10 ppm to −25 ppm regions. Adapted from Ref. [137] with permission.

FDX1 is a versatile electron mediator involved in multiple physiological processes such as donating electrons to cytochrome P450 enzymes as part of steroid hormone biosynthesis and vitamin D metabolism [134]. FDX2 (or FDX1L) is a newly characterized mitochondrial ferredoxin [135]. A deleterious mutation on FDX2 has been shown to be associated with a novel mitochondrial muscle myopathy, although the clinical phenotype is relatively mild [69]. Conflicting studies regarding the functions of these two ferredoxins have been reported. Shi and co-workers provided evidence that both FDX1 and FDX2 are important for Fe-S cluster biogenesis [136], whereas Sheftel et al. claimed that FDX1 is specifically involved in the production of steroid hormones and FDX2 is essential for the biosynthesis of heme A as well as Fe-S cluster assembly [135]. NMR has been used to investigate the interaction between FDX1/2 and other proteins in ISC machinery [132,137]. It was shown that FDX2 interacts with ISCU [132]. We assigned the backbone ^1^H-^15^N signals of the HSQC spectra of reduced and oxidized FDX1 and FDX2, and we followed changes in these NMR signals upon addition of (NIA)_2_. The results demonstrated that both reduced and oxidized FDX1 and FDX2 interact with the cysteine desulfurase complex. Mapping of the chemical shift perturbations onto the structures of the ferredoxins showed that highly conserved α3 helix and β4 strand close to the [2Fe-2S] clusters of the ferredoxins are the sites of interaction with the cysteine desulfurase (Figure 4A–D). Because the α3 helix has also been shown to bind FDXR [138], this suggests that ferredoxin (FDX1/FDX2) cannot be reduced by FDXR while bound to (NIA)_2_. NMR spectra indicated that the apo forms of both ferredoxins are disordered, and NMR titration studies revealed no interaction of either apo-ferredoxin with (NIA)_2_. The hyperfine ^1^H chemical shifts of FDX1 and FDX2 are similar (Figure 4E,F), indicating similar unpaired electron delocalization from the [2Fe-2S] cluster onto the protein ligands. In addition, we showed that both reduced FDX1 and FDX2 can provide electrons for the reduction of S^0^ and Fe-S cluster assembly. We also showed that, in comparison to FDX1, FDX2 binds (NIA)_2_ more tightly and supports more rapid in vitro Fe-S cluster assembly. Thus, FDX2 appears to be the predominant electron donor in mitochondrial Fe-S cluster biogenesis [137].

The *de novo* assembly of a Fe-S cluster on ISCU also requires a source of iron. The identity of the primary iron source in mitochondrial ISC machinery has remained unclear despite of intense research interest. Several candidates have been proposed, including frataxin (FXN) [139,140], iron importer mitoferrin1 [141], and the mitochondrial pool of labile iron [142]. 

FXN is a highly conserved small acidic protein that is expressed in tissues rich in mitochondria, such as heart, liver, and neurons [64,143]. Deficiency of FXN is associated with the neurodegenerative disease Friedreich ataxia, commonly resulting from a GAA trinucleotide repeat expansion in the FXN gene [64,144]. Several structures of FXN or its homologs have been obtained by solution NMR, which show very similar folds [145,146,147]. FXN has been shown to enhance Fe-S cluster assembly on ISCU by serving as an allosteric switch for NFS1 and to stimulate sulfur transfer from NFS1 to ISCU [90,148,149]. The bacterial frataxin homolog (CyaY), on the other hand, is a negative regulator and inhibits Fe-S cluster biosynthesis in *E. coli* [31,32]. It has been shown that the activation or inhibition of Fe-S cluster assembly is determined by the cysteine desulfurase rather than the frataxin homolog [150].

FXN has also been proposed to be iron donor owing to its ability to bind iron and transfer iron to ISCU for in vitro Fe-S cluster assembly [139,151]. Solution NMR studies showed that the conserved acid ridge of FXN, which consists of the α1 helix and β1 strand, forms the iron-binding site [151]. However, the discovery of an ISCU mutant in yeast that survives without frataxin [152,153,154] has cast doubt on the hypothesis that FXN serves as the primary iron donor. It has been suggested that FXN, rather than being the iron donor, controls the entry of iron to ISCU [155,156]. 

**Figure 5:**
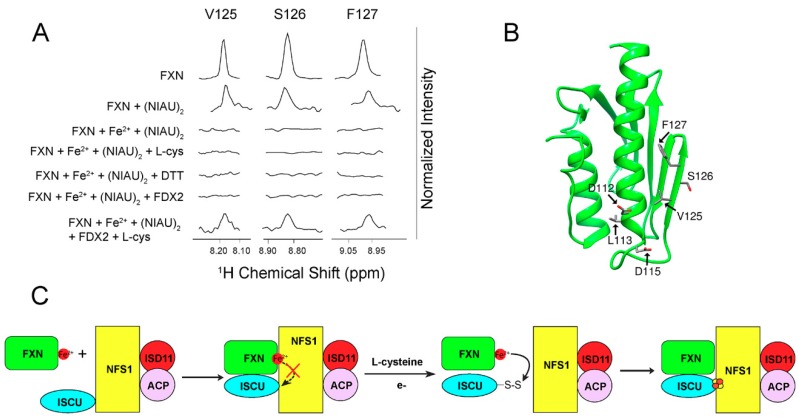
Results showing that Fe^2+^ bound to FXN remains bound when FXN interacts with (NIA)_2_ but becomes labile upon the addition of both l-cysteine and FDX2. (**A**) One-dimensional sections along the ^1^H-dimension from two-dimensional ^1^H-^15^N TROSY-HSQC peaks assigned to residues V125, S126 and F127 under conditions specified in the figure; (**B**) Structure of FXN (PDB: 1EKG) indicating the positions of the residues studied; (**C**) Schematic representation of the possible mechanism by which FXN controls iron entry into ISCU. Adapted from Ref. [156] with permission.

To investigate these questions, we followed changes in the ^1^H-^15^N HSQC NMR spectrum of [U-^15^N]-FXN upon binding (NIA)_2_ and/or Fe(II). The results revealed that both (NIA)_2_ and Fe(II) interact with the α1-β1 acidic ridge of FXN. Similar studies showed that ISCU binds to the β3-β5 sheet of FXN in an (NIA)_2_-dependent manner. By monitoring the ^1^H-^15^N HSQC signals of α1-β1 acid ridge in the HSQC spectra of FXN, we showed that FXN-Fe(II) binds to the (NIAU)_2_ complex without release of iron. Subsequent experiments demonstrated that Fe(II) was released from FXN only when both l-cysteine and a reductant (either FDX2 or DTT) were added; the addition of either alone failed to trigger release [156] (Figure 5). These results indicate that iron release from FXN is controlled such that it happens only after sulfur is mobilized for cluster assembly. Release of iron could be triggered by reaction of FXN-bound Fe(II) with the sulfur radical anion following its transfer from the cysteine desulfurase to ISCU; this reaction would yield S^2−^ and Fe(III), and, because we have shown that Fe(III) does not bind to FXN, would explain its release [156]. Alternatively, a conformational change occasioned by the binding of l-cysteine and an electron donor could account for iron release.

**Figure 6:**
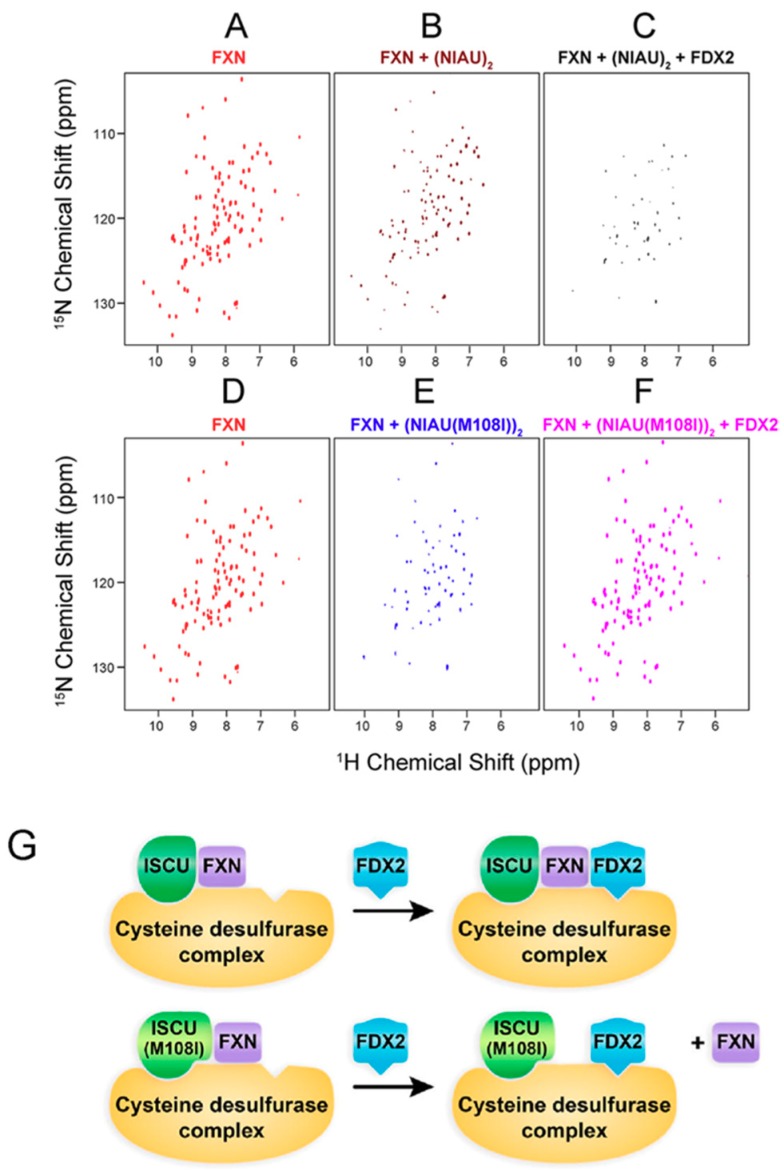
NMR evidence that FDX2 binds to (NIAUF)_2_ without displacement of FXN but that FDX2 added to (NIAU(M108I)F)_2_ displaces FXN. (**A**) ^1^H-^15^N TROSY-HSQC spectrum of [U-^15^N]-FXN; (**B**) ^1^H-^15^N TROSY-HSQC spectrum of [U-^15^N]-FXN after the addition of 0.5 subunit equivalent of unlabeled (NIAU)_2_; (**C**) ^1^H-^15^N TROSY-HSQC spectrum of [U-^15^N]-FXN after the addition of 0.5 subunit equivalent of unlabeled (NIAU)_2_ and 1.0 subunit equivalent of unlabeled FDX2; (**D**) ^1^H-^15^N TROSY-HSQC spectrum of [U-^15^N]-FXN; (**E**) ^1^H-^15^N TROSY-HSQC spectrum of [U-^15^N]-FXN after the addition of 0.5 subunit equivalent of unlabeled (NIAU(M108I))_2_; (**F**) ^1^H-^15^N TROSY-HSQC spectrum of [U-^15^N]-FXN after the addition of 0.5 subunit equivalent of unlabeled (NIAU(M108I))_2_ followed by the addition of 1.0 subunit equivalent of unlabeled FDX2; (**G**) Schematic summary of the NMR data. Adapted from Ref. [157] with permission.

The mechanism behind the FXN-bypassing ISCU mutant remains unclear [152,153,154]. We showed that when reduced FDX2 serves as the electron donor, FXN stimulates in vitro cluster assembly on wild-type ISCU; however, no stimulation by FXN was observed when clusters were assembled on ISCU(M108I), the human equivalent of the FXN-bypassing ISCU mutant [157]. Structural and mechanistic studies of the ISC core complex are challenging because the complex involves the dynamic association of several proteins [63]. NMR is one of the few techniques that is capable of probing weak, dynamic protein–protein complexes. By using NMR titration experiments, we showed that wild-type ISCU, FXN, and FDX2 all bind to (NIA)_2_, forming a large complex (NIAUFX)_2_. However, when wide-type ISCU was replaced with ISCU(M108I) in the (NIAUF)_2_ complex, the addition of FDX2 displaced FXN leading to (NIAUX)_2_ complex rather than (NIAUFX)_2_ [157] (Figure 6). The displacement of FXN by FDX2 explains why FXN fails to stimulate cluster assembly on ISCU(M108I). The mechanism behind this difference between wild-type ISCU and ISCU(M108I) remains to be determined. One clue, however, is our finding that ISCU(M108I) is fully structured, rather than metamorphic as is wild-type ISCU [157]. *E. coli* IscU contains an isoleucine at residue position 108, and thus the M108I mutation, makes human ISCU bacterial-like. In the *E. coli* system, CyaY was found to compete with Fdx for binding to the IscS-IscU complex [158,159]. In this case, CyaY succeeded in displacing Fdx thereby inhibiting the cluster assembly reaction. The yeast (*S. cerevisiae*) system resembles the human mitochondrial system in that ferredoxin (Yah1), scaffold protein (Isu1), and frataxin (Yfh1) bind simultaneously to the cysteine desulfurase to form a large complex [132]. Recent small angle X-ray scattering (SAXS) studies by Boniecki et al. also provide evidence for formation of (NIAUFX)_2_ [92]. These findings underline the critical differences between eukaryotic and prokaryotic ISC machineries, and they also suggest a mechanism to explain the strikingly different functions of frataxin in these two machineries. Another major difference between these two systems is that bacteria possess another iron-binding protein IscX (also known as YfhJ), which has no human homolog [160]. IscX binds Fe(II) and has been proposed to be the iron donor in bacterial ISC system [161]. It has been shown that IscX competes with CyaY for the binding to IscS [122,161,162]. A recent study suggests that IscX can rescue the Fe-S cluster assembly rate that is inhibited by CyaY by displacing CyaY from IscS-IscU complex, and that the rescuing effect is under the control of iron: the effect is strong at low iron level and negligible at high iron level [162]. These studies together suggest CyaY and IscX serve as dual regulators of the bacterial ISC system, which is strikingly different from the proposed function of FXN in the human ISC system. 

**Figure 7:**
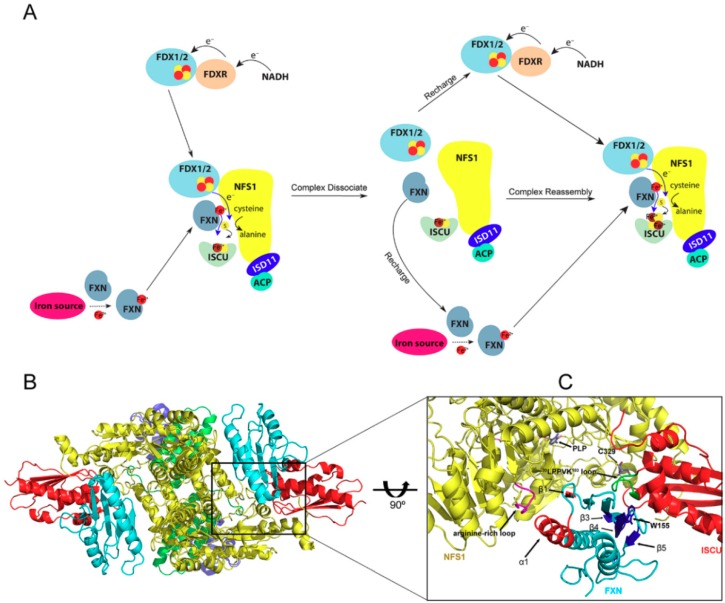
Proposed steps of Fe-S cluster assembly on ISCU and a structural model of (NIAUF)_2_. (**A**) Schematic representation of the steps leading to Fe-S cluster assembly on human ISCU. It starts with the recruitment of ISCU by (NIA)_2_ to form (NIAU)_2_. Then, reduced ferredoxin (X) and Fe^2+^-FXN (F) bind to yield (NIAUFX)_2_. (NIA)_2_ converts l-cysteine to l-alanine to generate sulfane sulfur. An electron from reduced ferredoxin converts the sulfane sulfur to a radical anion (−S^•^), which is transferred to one of the cysteine residues of ISCU, and an electron transferred from Fe^2+^ to the radical anion leads to the formation of -S^2−^ and Fe^3+^. In the next stage, FXN and oxidized ferredoxin dissociate from the (NIAUFX)_2_ complex. Ferredoxin is reduced by ferredoxin reductase (FDXR), and Fe^2+^-FXN is regenerated with Fe^2+^ from a, yet to be identified, mitochondrial iron protein. Then reduced ferredoxin and Fe^2+^-FXN bind back to the (NIAU)_2_ complex, and the cycle is repeated to complete the assembly of a [2Fe-2S] cluster; (**B**) A structural model of (NIAUF)_2_ built by the HADDOCK server by combining NMR and XL-MS restraints with the X-ray structures of (NIAU)_2_ (PDB: 5WLW) and FXN (PDB: 1EKG); (**C**) Expanded view of the boxed region in (**B**). The regions of the FXN model colored red and blue indicate its binding interfaces with NFS1 and ISCU, respectively, as identified from NMR studies. The arginine-rich loop on NFS1 (magenta) and the “^99^LPPVK^103^” loop on ISCU (green) are involved in the interaction with FXN. The inset was rotated 90° for better view of the protein interaction interfaces of the complex. See Ref. [129] for details.

The current model of the mechanism of mitochondrial Fe-S cluster assembly consists of the following steps (Figure 7A). It starts with the recruitment of ISCU by (NIA)_2_ to form (NIAU)_2_. Then, reduced ferredoxin (X) and Fe^2+^-FXN (F) bind to yield (NIAUFX)_2_ [132,157]. By combining constraints from NMR, SAXS and XL-MS as input to HADDOCK [156,163], we generated a structural model of (NIAUF)_2_ (Figure 7B,C) [129]. In this model, FXN is found to bind in a cleft between ISCU and NFS1, the α1-β1 site of FXN interacts with the conserved arginine-rich loop “^271^RRRPRVR^277^” of NFS1, and the β3-β5 sheet interacts with the conserved “^99^LPPVK^103^” motif of ISCU [164]. Binding of FXN opens up the active site of NFS1 allowing the entry of l-cysteine, which upon conversion to l-alanine generates S^0^ bound to the active site cysteine (C381) of NFS1. An electron from reduced ferredoxin converts the bound sulfur to a radical anion (-S^•^), which is transferred to one of the cysteine residues of ISCU (the identity of that residue remains in question [122,148,165,166]). Then an electron transferred from Fe^2+^ to the radical anion leads to the formation of -S^2−^ and Fe^3+^. In the next stage, FXN and oxidized ferredoxin are released. Ferredoxin is reduced by ferredoxin reductase (FDXR), which binds to the same surface of ferredoxin that binds NFS1 [137,138], and Fe^2+^-FXN is regenerated with Fe^2+^ from a, yet to be identified, mitochondrial iron protein. Then reduced ferredoxin and Fe^2+^-FXN bind back to the (NIAU)_2_ complex, and the cycle is repeated to complete the assembly of a [2Fe-2S] cluster. Future studies are needed to test the key steps of this model. 

## 3. [2Fe-2S] Cluster Transfer as Studied by NMR Spectroscopy

The second step of mitochondrial ISC machinery is the transfer of the nascent Fe-S cluster assembled on ISCU to a recipient Fe-S protein. This process is facilitated by the mitochondrial HSP70-HSC20 chaperone–cochaperone system in an ATP-dependent manner [167,168] (Figure 1). The homologous chaperone and cochaperone in the *E. coli* ISC system are HscA and HscB, respectively. Mitochondrial HSP70 (also known as mortalin or HSPA9) is a multifunctional protein involved in many cellular processes such as stress response, control of cell proliferation, and inhibition/prevention of apoptosis [169,170]. The specificity of HSP70 toward different cellular functions is driven by its interaction with J-protein cochaperones [171,172]. HSC20 is the cochaperone involved in Fe-S cluster transfer [173]. Recent studies suggest that HSC20 facilitates Fe–S cluster delivery to target proteins by recognizing the “LYR” motif in specific recipient Fe–S proteins or accessory factors [174,175]. HSP70 recognizes the conserved “LPPVK” loop of ISCU [176]; however, this loop is not exposed in the S-state of ISCU. Solution NMR studies showed that HSP70 binds to the D-state of ISCU, whereas HSC20 binds to the structured state, and the β-sheet of ISCU (S-state) forms the binding interface for HSC20 [84,96]. The mechanism of transfer of holo-ISCU from the (NIAU)_2_ complex to the co-chaperone is unknown. A possible mechanism is that HSC20 displaces cysteine desulfurase from ISCU because their binding to ISCU is mutually exclusive [173]. The HSC20-holo-ISCU complex then interacts with the ATP-bound state of HSP70. Both ISCU and HSC20 simulate the ATPase activity. ATP hydrolysis induces a conformational change of HSP70, and ISCU undergoes a conformation change (likely from the S- to the D-state) that makes its “LPPVK” loop accessible to HSP70 and stimulates release of the [2Fe-2S] cluster to a recipient protein [167,177]. The nucleotide exchange factor (NEF) then binds to HSP70 and catalyzes the exchange of ADP for ATP to set up the subsequent cycle [178]. 

Recent studies have shown that Fe-S cluster release from ISCU can involve the single-domain monothiol glutaredoxin 5 (GLRX5, Grx5 in yeast) [131]. A member of monothiol glutaredoxin family with a signature CGFS active site, GLRX5 has been characterized as an intermediate Fe-S cluster carrier involved in cluster transfer downstream of ISCU [39]. *E. coli* Grx5 can receive a [2Fe-2S] cluster from IscU, and the presence of HscA-HscB leads to a 700-fold increase in the transfer rate [179]. In yeast, Grx5 has been shown to interact with the Hsp70 chaperone Ssq1 at a site close to but not overlapping with the Isu1 binding site. The proximity of Isu1 and Grx5 on the Hsp70 chaperone allows for rapid Fe-S cluster transfer from Isu1 to Grx5 [38]. A crystal structure has shown that each of two GLRX5 molecules provides one cysteinyl ligand to the bridging [2F-2S] cluster and that the remaining two thiolate ligands come from glutathione (GSH) molecules bound to each GLRX5 [180]. Solution NMR studies show that GLRX5 is monomeric in solution and dimerizes upon cluster binding [181]. Although its cluster binding site is less ordered, the backbone conformation of the solution NMR structure of apo-GLRX5 [181] is similar to that of the GLRX5 subunit in the X-ray structure of the holo-protein [180]. Interestingly, ^1^H-^15^N IR-HSQC-AP and paramagnetic ^13^C-NMR experiments of [2Fe-2S]-GLRX5 showed two set of peaks around the Fe-S cluster binding site, indicating that two dimeric [2Fe-2S]-GLRX5 species exist in solution [181]. Monothiol glutaredoxins and A-type proteins have been shown to be partners in Fe-S cluster trafficking [182]. Using NMR titration experiments, Banci et al. observed unidirectional Fe-S cluster transfer from GLRX5 to A-type Fe-S cluster carriers, ISCA1 and ISCA2. ^15^N-NMR relaxation experiments indicate that ISCA1 and ISCA2 receive the [2Fe-2S] cluster from [2Fe-2S]-GLRX5 in their hetero-dimeric state [181]. 

Recent studies by Maio et al. suggest an alternative mechanism of Fe-S cluster transfer from ISCU that is independent of intermediate carriers such as GLRX5 [183]. Maio et al. showed that HSC20 can guide the target of specific Fe–S recipient proteins for cluster delivery by binding to a consensus LYR sequence present in specific proteins [175]. One example is succinate dehydrogenase subunit b (SDHB) in respiratory complex II, which contains three LYR motifs close to the Fe-S cluster binding sites. Maio et al. showed that HSC20 was able to guide the insertion of [2Fe-2S] cluster from holo-ISCU to SDHB by recognizing the LYR motifs [175]. In another recent study, Maio et al. demonstrated that HSC20 could guide the insertion of [2Fe-2S] cluster from holo-ISCU to a Rieske protein UQCRFS1 in complex III by binding to the LYR sequence in LYRM7, which forms an intermediate assembly complex with UQCRFS1 [174]. 

## 4. Maturation of [4Fe-4S] Cluster by NMR 

[4Fe-4S] clusters are the most commonly used Fe-S cluster in cells. Multiple in vitro studies have shown that [4Fe-4S] can be assembled on ISCU [155,184,185,186]. However, recent in vivo studies by the Lill and other groups suggest that ISCU can only form a [2Fe-2S] cluster and that the maturation of [4Fe-4S] clusters in mitochondria requires a set of other mitochondrial proteins, including two A-type carrier proteins (ISCA1 and ISCA2) and IBA57, a protein currently with no known function except for its involvement in the maturation of Fe-S proteins containing [4Fe-4S] clusters [44,45]. A recent study using a mouse model suggested that only ISCA1 is required for the maturation of mitochondrial [4Fe-4S] clusters [43]. Defects in ISCA1, ISCA2 and IBA57 have been associated with numerous mitochondrial diseases now categorized as multiple mitochondrial dysfunctions syndromes MMDS3 (IBA57) [80,81,187], MMDS4 (ISCA2) [79], and MMDS5 (ISCA1) [77,78]. A common phenotype of these MMDS diseases is the deficiency in mitochondrial [4Fe-4S] proteins, such as lipoic acid synthase (LAS), a strong indication of their functions in mitochondrial [4Fe-4S] cluster maturation. Solution NMR studies by Brancaccio et al. provide direct in vitro evidence supporting the role of ISCA1-ISCA2 in [4Fe-4S] cluster maturation [188]. ^15^N-NMR relaxation experiments showed that apo-ISCA2 exists in solution as a homodimer. ESI-MS, EPR, and paramagnetic ^1^H-NMR studies showed that, whereas the “as purified” ISCA2 dimer contains a [2Fe-2S] cluster, the chemically reconstituted dimeric ISCA2 contains a [4Fe-4S] cluster. The nature of the cluster bound to ISCA2 can be altered by changing the redox conditions. NMR titration experiments showed that ISCA2 and ISCA1 form a heterodimeric complex. Mapping of the chemical shift perturbations suggested that the ISCA2-ISCA1 heterodimer interface closely resembles that of (ISCA2)_2_ homodimer. By ^1^H-^15^N HSQC NMR and UV/vis studies, the authors demonstrated that [2Fe-2S]-GLRX5 can transfer its cluster to ISCA2-ISCA1 and that the cluster transfer occurs via a weak, transient interaction between [2Fe-2S]-GLRX5 and ISCA2-ISCA1. NMR, EPR, and UV/vis data all identified the new cluster on ISCA2-ISCA1 as [4Fe-4S], indicating that the ISCA2-ISCA1 heterodimer is capable of converting two [2Fe-2S] clusters to a [4Fe-4S] cluster. It remains unclear what role IBA57 plays in the maturation of [4Fe-4S] clusters, although IBA57 has been shown to interact with ISCA2 and to potentially form a hetero-trimeric complex with ISCA1-ISCA2 [43,45]. The cluster transfer from GLRX5 to ISCA2-ISCA1 likely involves a chaperone–cochaperone system, although it remains to be seen how it functions in the cellular environment. In addition, a biological electron donor, such ferredoxin, may also be needed to reduce two [2Fe-2S] clusters to a [4Fe-4S] cluster. A recent NMR study by Brancaccio et al. demonstrated that the [4Fe-4S] cluster maturation on ISCA2-ISCA1 is impaired by Cu(I): Cu(I) can bind ISCA2-ISCA1 tightly and prevent ISCA2-ISCA1 from receiving Fe-S clusters from holo-GLRX5. The authors suggested that impaired [4Fe-4S] cluster maturation may explain cellular Cu(I) toxicity [189]. 

## 5. Trafficking of [4Fe-4S] Cluster Studied by NMR 

Following the maturation of [4Fe-4S] clusters as described above, the trafficking of these [4Fe-4S] clusters in mitochondria is facilitated by late-acting ISC targeting factors or intermediate [4Fe-4S] cluster carriers, such as NFU1 and NUBPL, which receive [4Fe-4S] clusters from [4Fe-4S]-ISCA1-ISCA2 complex and deliver them to downstream recipient proteins [47,48,190,191,192,193]. 

One of these late-acting factors is NFU1, a protein that binds a [4Fe-4S] cluster and was initially thought to be an alternative scaffold protein in the ISC machinery [194]. Clinical and genetic studies of patients of multiple mitochondrial dysfunctions syndromes 1 (MMDS1) identified mutations in the gene for NFU1 [47,48,195]. The biochemical phenotype suggested deficiency of a subset of [4Fe-4S] proteins such as lipoic acid synthase (LIAS) and succinate dehydrogenase (SDH). NFU1 was thus characterized as a late-acting factor required for the maturation of a subset of [4Fe-4S] proteins. Studies in yeast showed that NFU1 binds ISCA and [4Fe-4S] client proteins [46]. NFU1 consists of two domains: a highly conserved C-terminal domain (CTD) and a less conserved N-terminal domain (NTD). The structures of CTD and NTD were determined separately by solution NMR spectroscopy [196]. The NTD has a βββαββα fold with an additional short a turn α1′ between strand β3 and helix α1; the CTD has a αββαβ fold with a kink in the middle of helix α1. In both domains, the β strands form an antiparallel β sheet, and the two helices pack on one side of the β sheet to form a two-layer sandwich topology. The conserved CXXC-containing-motif that serves as the Fe-S cluster ligands is located on a flexible loop between β2 and α2 of CTD [196]. Small angle X-ray scattering (SAXS) data indicated that full-length NFU1 adopts a dumbbell-shaped structure, with CTD and NTD connected by a flexible linker region, similar to that of *Arabidopsis thaliana* NFU protein [196,197]. NFU1 contains only two cysteine residues, which are located in the CTD and are essential for activity. Thus, two CTD molecules are needed to coordinate the [4Fe-4S] cluster. 

**Figure 8:**
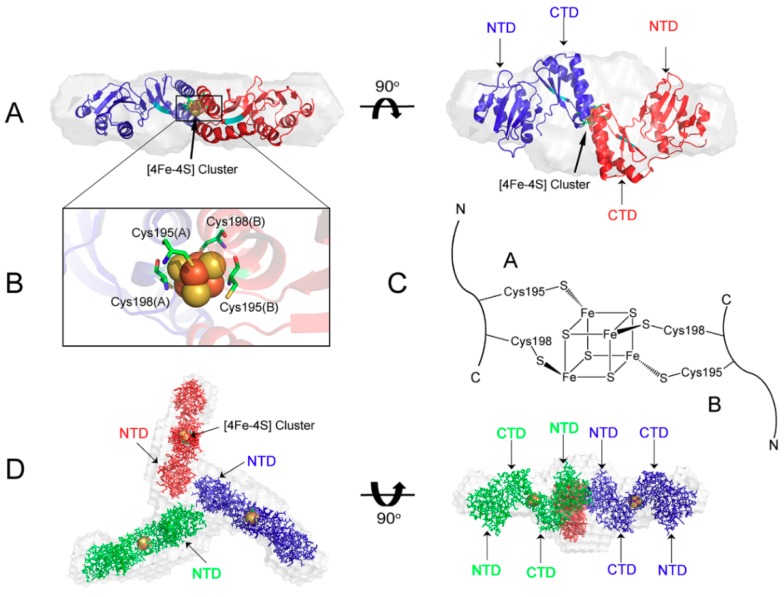
Structural model of [4Fe-4S]-NFU1. (**A**) Rigid body reconstructed model of the [4Fe-4S] cluster-bound dimer of NFU1 superimposed onto the dummy atom model reconstructed from the SAXS data for dimeric apo-NFU1; (**B**) Expansion of the region of the [4Fe-4S] cluster; (**C**) Configuration of cluster ligation consistent with the SAXS results; (**D**) The rigid body reconstructed model of the trimer of cluster-containing dimers of NFU1 superimposed onto the dummy atom model reconstructed from the SAXS data for holo-NFU1. The tripartite binding region is formed by the N-terminal domain of one NFU1 subunit of each of three NFU1-[4Fe-4S]-NFU1 units. The model is consistent with NMR studies. Adapted from Ref. [196] with permission.

We determined from NMR diffusion, SAXS, size-exclusion chromatography (SEC), and analytical ultracentrifugation (AUC) measurements that [4Fe-4S]-NFU1 made by enzymatic reconstitution exists in solution as a hexamer [196]. According to a SAXS-based ab initio model, the structure is a Y-shaped complex composed of three [4Fe-4S]-(NFU1)_2_ units. Each of the three legs consists of two NFU1 molecules whose CTDs ligate the [4Fe-4S] cluster. One NTD of each leg is free and the other NTD participates in a tripartite complex (Figure 8). This model predicts two sets of NTD NMR peaks, one for the free ends and one for the complexed end. This prediction was confirmed by NMR spectra [196]. We speculate that the bundling of three [4Fe-4S] clusters in a single aggregate may offer an efficient mechanism for cluster delivery to recipient proteins. 

A few studies have investigated cluster transfer from NFU1 to a recipient protein. Gao et al. showed by monitoring circular dichroism (CD) spectra changes that *Arabidopsis thaliana* NFU2 can transfer a [4Fe-4S] cluster to APR1 [198]. *E. coli* holo-NfuA was capable of transferring a [4Fe-4S] cluster to lipioyl synthase (LipA) and activating LipA activity [199]. We showed that holo-NFU1 can transfer its [4Fe-4S] cluster to mitochondrial apo-aconitase (AcnA) and activate its AcnA activity. ^1^H-^15^N HSQC NMR spectra of NFU1 before and after cluster delivery to AcnA revealed that NFU1 became monomeric after transferring the Fe-S cluster to AcnA. Our NMR data also suggest that the cluster transfer process likely adopts a “hit-and-run” mechanism as no interaction was detected by NMR between apo-NFU1 and holo-AcnA after cluster transfer [196]. 

[4Fe-4S] cluster maturation in mitochondria also requires BOLA family proteins. BOLA-like proteins are known to partner with monothiol glutaredoxins (Grxs) and mediate Fe-S cluster delivery and cellular iron regulation [200]. The human proteome contains three BOLA proteins, namely BOLA1, BOLA2, and BOLA3. BOLA1, and BOLA3 are localized to mitochondria, whereas BOLA2 is localized to cytosol and involved in CIA pathway. Deficiency of BOLA3 is associated with mitochondrial disease MMDS2 [48]. Depletion of BOLA1 in cultured human cells leads to increased mitochondrial protein thiol oxidation and changes in mitochondrial morphology [201]. Recent studies have confirmed that both BOLA1 and BOLA3 are important for mitochondrial [4Fe-4S] cluster maturation and that they play overlapping, but not entirely identical, roles in this process [46,202]. The structures of BOLA1 and BOLA3 have been solved recently by solution NMR [202]. The NMR structures of BOLA1 and BOLA3 showed similar αββαβ folds, with the three β strands forming a β-sheet (β1↓β2↑β2↑) and the two helices packing on one side of the β sheet to form a two-layer sandwich topology. Both are structurally similar to other BOLA-like proteins from *Mus musculus* and *Arabidopsis thaliana* [203,204]. The key differences between BOLA1 and BOLA3 are in the loop between β1 and β2, which contains several key residues that may potentially bind Fe-S clusters. NMR titration showed that both BOLA1 and BOLA3 bind apo-GLRX5 and holo-GLRX5. NMR relaxation and thermophoresis results showed that both BOLA proteins form a 1:1 complex with apo-GLRX5 with *K*_d_ about 3 µM. Mapping of the NMR chemical shift perturbations accompanying the formation of complexes onto the structures of apo-GLRX5, BOLA1, and BOLA3 revealed that helix α2, β-sheet and an invariant His residue H96 of both BOLA proteins are involved in their interaction with GLRX5. The authors further studied interaction between [2Fe-2S]-GLRX5 and BOLA proteins. Chemical shift perturbation analysis showed that the NMR signals from putative Fe-S cluster binding sites on the BOLA proteins were affected by the addition of holo-GLRX5, whereas NMR signals from the GSH binding site and surrounding residues of GLRX5 were affected by the addition of BOLA proteins. The ^1^H-^15^N HSQC and ^15^N relaxation data from GLRX5-BOLA complexes were consistent with the formation of 1:1 heterodimeric, [2Fe-2S]-bridged, GLRX5-BOLA complexes [202]. UV/vis, CD, EPR, and NMR data suggested that the BOLA1-GRX5 complex coordinates a reduced Rieske-type [2Fe-2S]^1+^ cluster, whereas the BOLA3-GRX5 complex coordinates an oxidized, ferredoxin-like [2Fe-S]^2+^ cluster. The [2Fe-2S]-BOLA1-GRX5 complex is formed in preference to the [2Fe-2S]-BOLA3-GRX5 complex, as the result of its higher cluster binding affinity [205]. 

Although both BOLAs interact with GLRX5 and can form heterodimeric holo-complexes, it has also been shown that BOLA1 is better at stabilizing the [2Fe-2S] cluster on GLRX5 than BOLA3, indicating the potential functional differences between the two BOLA proteins as a consequence of different stability, redox potential and solvent accessibility properties of the [2Fe-2S] clusters [205]. Several lines of evidence indicate that NFU1 and BOLA3 act together as late-acting factors for [4Fe-4S] cluster maturation in LIAS and SDH: a) mutations on NFU1 (MMDS1) and BOLA3 (MMDS2) have similar clinic and biochemical phenotypes such as severe epileptic encephalopathy and dilated cardiomyopathy, severely reduced activities of pyruvate dehydrogenase complex (PDHc) and α- ketoglutarate dehydrogenase complex (α-KGDH), due to reduced lipoylation of the E2 subunits of these enzymes, and deficiency of respiratory chain complexes I, II and III [47,48]; b) NFU1 interacts with BOLA3; and c) overexpression of Nfu1 leads to increased level of Bola3 expression in yeast cells [46,202]. However, the precise function of BOLA3 in this process remains elusive. In addition, the physiological function of GLRX5-BOLA1 is a mystery. It could play a role in mitochondrial iron sensing and regulation, similar to that of the yeast cytosolic Grx5-Bol2 complex [200,206], or it may assist GLRX5 in [2Fe-2S] delivery to the ISCA1-ISCA2 complex [141].

## 6. CIA Machinery as Studied by NMR Spectroscopy

The biogenesis of cytosolic and nuclear Fe-S proteins involves intimate coordination between the mitochondrial ISC machinery and the CIA system. The CIA system utilizes a sulfur-containing compound (X-S) synthesized by mitochondrial ISC machinery and exported via an ATP binding cassette (ABC) transporter (Atm1 in yeast, ABCB7 in human) located in the mitochondrial inner membrane [42,207,208,209]. The identity of X-S is still undetermined, however, a recent study using size-exclusion chromatography combined with inductively coupled plasma mass spectrometry (SEC-ICP-MS) suggested that the X-S species has a molecular mass between 700 and 1000 Da [210]. 

**Figure 9:**
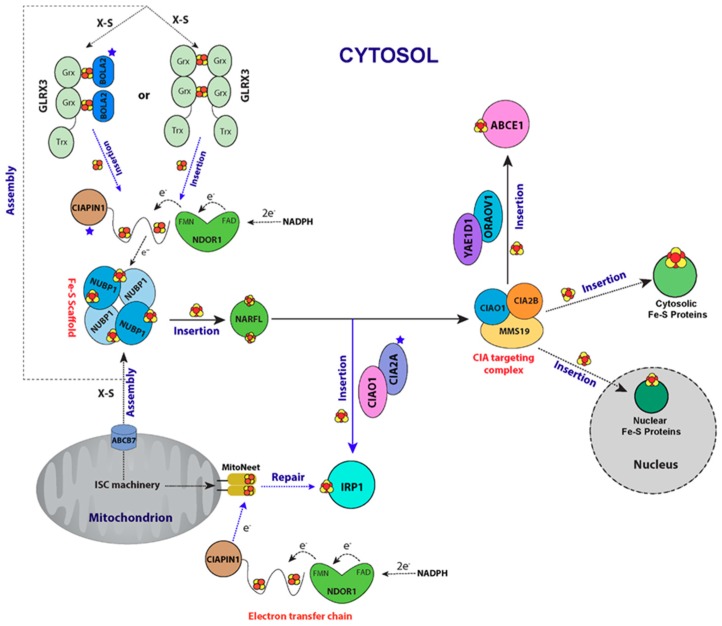
Schematic representation of the current model of human cytosolic Fe-S cluster assembly (CIA machinery). The CIA machinery requires the mitochondrial ISC assembly machinery, which synthesizes an unidentified sulfur-compound (X-S) that is exported from mitochondria by ABCB7. The X-S compound might be used to assemble [4Fe-4S] clusters on the (NUBP1-NUBP2)_2_ heterodimeric complex and assembly of [2Fe-2S] clusters on GLRX3 and GLRX3-BOLA2. The electrons needed for [4Fe-4S] clusters assembly on the (NUBP1-NUBP2)_2_ are provided by the electron transfer chain composed of NADPH, NDOR1, and anamorsin. [2Fe-2S]-(GLRX3)_2_ homodimer or the [2Fe-2S]_2_-GLRX3-BOLA2_2_ heterotrimer are capable of transfer Fe-S cluster to anamorsin. [4Fe-4S] clusters on (NUBP1-NUBP2)_2_ are subsequently transferred to the intermediate carrier NARFL, which transfers them into cytosolic and nuclear [4Fe-4S] target proteins. The latter process is assisted by the CIA targeting complex CIAO1-CIA2B-MMS19. Other late-acting factors may be required for the maturation of certain cytosolic Fe-S proteins. The YAE1D1-ORAOV1 complex is required for the maturation of ABCE1, and the CIA2A-CIAO1 complex is required for the maturation of cytosolic aconitase IRP1. MitoNEET has been shown to be capable of repairing an Fe-S cluster on IRP1 damaged under oxidative stress conditions. NDOR1-anamorsin complex is capable of reducing the Fe-S cluster on mitoNEET to its dormant [2Fe-2S]^+^ state. The maturation of mitoNEET is shown to be dependent on mitochondrial ISC machinery. The blue stars denote the proteins (or the protein homologs) for which the structures were obtained by NMR, and blue arrows denote the processes that were clarified by NMR.

To date, 13 CIA components have been identified (Table 2). The current working model of CIA can be divided into two major steps (Figure 9). First, a transiently bound [4Fe-4S] cluster is assembled on a scaffold protein complex consisting of two P-loop NTPase proteins, namely NUBP1 (Nbp35 in yeast) and NUBP2 (Cfd1 in yeast) [211,212]. In the second step, the [4Fe-4S] cluster on the NUBP1-NUBP2 scaffold is transferred to dedicated apo-proteins such as IRP1 and ABCE1. This transfer reaction is mediated by the iron-hydrogenase-like protein NARFL (also known as IOP1, Nar1 in yeast) [213], the CIA targeting complex comprises CIAO1, CIA2B (yeast Cia1 and Cia2) [214,215], and MMS19 (yeast Mms19) [216,217]. Other late-acting factors that are required for the insertion of Fe-S cluster to specific apo-protein targets have also been identified. CIA2A has been shown to be a late-acting factor required for the maturation of cytosolic IRP1, a key factor for cellular iron homeostasis [215]. The structure of monomeric CIA2A have been obtained by solution NMR; two different X-ray structures of dimeric CIA2A forms have also been reported [218,219]. In a recent study, by using NMR, UV/vis and EPR, Maione et al. showed that CIA2A and CIAO1 form a heterodimeric species CIA2A-CIAO1, which can bind a [4Fe-4S] cluster. The CIA2A alone is incapable of binding any type of Fe-S cluster. The [4Fe-4S] cluster on CIA2A-CIAO1 complex can be transferred to apo-IRP1 to generate active form of IRP1 [220]. Two other CIA proteins, named ORAOV1 and YAE1D, have recently been identified, and shown to be late-acting factors required for the maturation of ABCE1 [221]. CIA machinery has been linked with DNA metabolism and genome stability [207]. For example, the CIA protein MMS19 is required for Fe-S transfer to enzymes involved in for DNA metabolism. Mutations in MMS19 cause genome instability, and the phenotypes include defects in methionine synthesis, sensitivity to genotoxic stress, and extended telomeres [217,222]. It is worth mentioning that a radically different mechanism regarding the synthesis and transfer of Fe-S clusters in cytosol has recently been proposed: Kim et al. have shown that Fe-S clusters can be synthesized *de novo* by cytosolic ISCU and NFS1 (cISCU and cNFS1) and that Fe-S cluster insertion into recipient proteins is mediated by cytosolic HSC20 (cHSC20) and CIA targeting complex (CIAO-CIA2B-MMS19), with cHSC20 recognizing the “LYR” motif on CIAO [223]. 

Electrons needed for the first step of the CIA pathway (Fe-S cluster assembly on the NUBP1-NUBP2 scaffold protein complex) are provided by a dedicated electron transport chain consisting of NADPH, NDOR1 (Tah18 in yeast), and anamorsin (also known as CIAPIN1, Dre2 in yeast) [224,225]. Human NDOR1, which belongs to the family of diflavin reductases, consists of two domains: The first binds FMN, and the second binds FAD and NADPH [225]. Anamorsin also consists of two domains: an N-terminal domain (NTD) and a C-terminal cytokine-induced apoptosis inhibitor 1 domain (CIAPIN1 domain), which contains two highly conserved cysteine-rich motifs (CX8CX2CXC and CX2CX7CX2C), and the two domains are connected by a flexible linker [230]. Banci et al. solved the structure of the NTD of anamorsin by solution NMR and showed by UV/Vis, EPR and ^1^H paramagnetic NMR experiments that the CIAPIN1 domain binds a [2Fe-2S] cluster through its first cysteine-rich motif [230]. CIAPIN1 domain binds another Fe-S cluster through its second cysteine-rich motif, although the identity of this second cluster is still the subject of debate [231,232]. Banci et al. used NMR spectroscopy to investigate the structural properties of the CIAPIN1 domain. ^15^N NOE and ^15^N R2/R1 NMR experiments indicated that CIAPIN1 is largely disordered [224]. Using NMR titration coupled with ^1^H-^15^N HSQC experiments, Banci et al. showed that the disordered [2Fe-2S] cluster binding site of the CIAPIN1 domain interacts with helices α2-α4 of the FMN-binding domain of NDOR1 and that the interaction is not dependent on the presence of a [2Fe-2S] cluster. The interaction mode enables electron transfer from the FMN-binding domain of NDOR1 to anamorsin. Banci et al. observed electron transfer from the reduced FMN-binding domain of NDOR1 to the [2Fe-2S]^2+^ cluster on the CIAPIN1 domain of anamorsin [224]. 

The CIA pathway also requires the function of a multidomain CGFS monothiol glutaredoxin (GLRX3) and a BOLA family protein (BOLA2). In yeast, Grx3 has been shown to interact with Fra2 (yeast BOLA2 homolog), which is required for ion sensing [200,206,233]. It has been shown that human GLRX3 and BOLA2 form a [2Fe-2S] cluster-bridging complex [226,234]. Furthermore, *in vivo* experiments by Frey et al. show that the GLRX3-[2Fe-2S]-BOLA2 complex can interact with the anamorsin-NDOR1 complex to serve as an Fe-S cluster chaperone in CIA machinery by transferring [2Fe-2S] clusters to the anamorsin-NDOR1 complex [226]. GLRX3 consists of three domains: one N-terminal Trx domain and two Grx domains (GrxA and GrxB), with each Grx domain binding a glutathione-coordinated [2Fe-2S] cluster via protein dimerization [226,234]. Solution NMR titration experiments by Banci et al. showed that the N-terminal Trx domain of GLRX3 interacts with the well-structured N-terminal domain of anamorsin, and that the interaction is stabilized by the unstructured linker region of anamorsin. The authors further showed by that [2Fe-2S] cluster on GLRX3 can be transferred to anamorsin and that the GLRX3-anamorsin complex binds two [2Fe-2S] clusters [235]. Using ^1^H-^15^N HSQC NMR, ^15^N *R*_2_/*R*_1_ NMR relaxation, and analytical gel filtration experiments, Banci et al. further showed that both Grx domains of GLRX3 interact simultaneously with BOLA2 to form a GLRX3:BOLA2_2_ complex with two bridging [2Fe-2S] clusters between GrxA-BOLA2 and GrxB-BOLA2. Chemical shift perturbation mapping revealed that the α3 helix and the β3 strand on BOLA2 interact with the [2Fe-2S] cluster-binding sites and their surrounding regions of GrxA and GrxB [228]. Banci et al. further showed that [2Fe-2S]_2_-GLRX3-BOLA2_2_ can donate a [2Fe-2S] cluster to anamorsin, as consistent with the in vivo findings that the GLRX3-BOLA2 complex serves as the chaperone that transfers a [2Fe-2S] cluster to anamorsin [226,228].

Recent NMR studies suggest a potential relationship between CIA machinery and mitoNEET. MitoNEET is a ~17 kDa [2Fe-2S] protein anchored on the outer mitochondrial membrane by a single transmembrane helix; its soluble region is located in the cytosol. MitoNEET, which is a diabetes and cancer drug target, plays a role in mitochondrial energy regulation, autophagy, redox sensing, and cell survival [236,237,238]. The crystal structure of MitoNEET shows that it forms a dimer, with each subunit containing a Rieske-type [2Fe-2S] cluster coordinated by side chains from three cysteine and one histidine residue [239]. A recent study showed that the maturation of mitoNEET is dependent on the mitochondrial ISC machinery and that mature mitoNEET can repair a damaged Fe-S cluster on cytosolic IRP1, a key regulator of cellular iron homeostasis [240]. By using ^1^H-^15^N SOFAST-HMQC NMR experiments, Ferecatu et al. demonstrated that mitoNEET can cycle between a well folded holo-form and a highly disordered apo-form just by insertion/disassembly/reinsertion of the Fe-S cluster [240]. NMR and UV/vis data by Golinelli-Cohen et al. show that mitoNEET can transfer the [2Fe-2S] cluster to a recipient protein. The transfer of [2Fe-2S] cluster depends on the redox state of the [2Fe-2S] cluster on mitoNEET. While the reduced [2Fe-2S]^+^ cluster is extremely stable and cannot be released, the oxidized [2Fe-2S]^2+^ cluster is labile and can be transferred to an apo-protein [241]. It is unclear what system regulates the redox states of mitoNEET. One possibility is the anamorsin-NDOR1 redox complex [242]. By using UV/vis spectroscopy, Camponeschi et al. showed that the FMNH^•−^-NDOR1-[2Fe-2S]^+^-anamorsin complex can reduce oxidized [2Fe-2S]^2+^-mitoNEET. The authors further demonstrated by ^1^H-^15^N HSQC NMR experiments coupled with protein titration that mitoNEET interacts with anamorsin but not NDOR1 in the FMNH^•−^-NDOR1-[2Fe-2S]^+^-anamorsin complex. Mapping of the chemical shift changes of mitoNEET revealed that the [2Fe-2S] cluster binding site of mitoNEET is most affected by binding to anamorsin [243]. This study provided in vitro evidence of a potential connection between the CIA system and mitoNEET. Overall, these NMR studies suggest a role for mitoNEET as a Fe-S repair protein for cytosolic IRP1 under oxidative stress. The Fe-S cluster repair is based on a redox switch mechanism. Under oxidative stress conditions, the [2Fe-2S] cluster of mitoNEET becomes oxidized and labile and can be released for IRP1 repair. Once oxidative stress is not present, the Ndor1/anamorsin complex of the CIA machinery reduces mitoNEET back to its dormant [2Fe-2S]^+^ form [240,241,243]. The finding that the maturation of the [2Fe-2S] cluster is dependent on mitochondrial ISC machinery [240] provides valuable insight into the intimate connection between the mitochondrial ISC machinery and the cytosolic CIA machinery. 

## 7. Conclusions 

Eukaryotic Fe-S cluster biogenesis is a complicated process that involves multiple factors forming intricate protein–protein interaction networks. Many of the protein–protein interactions are transient and weak, which make their detection and characterization challenging. In the past decade, solution NMR has contributed tremendously to the field of Fe-S cluster biogenesis. Solution NMR techniques have helped elucidate many key processes, including Fe-S cluster assembly and transfer, [4Fe-4S] maturation, and cytosolic Fe-S cluster biosynthesis. NMR is especially advantageous in studying the weak and transient protein–protein interactions that are prevalent in Fe-S cluster biogenesis. Although much progress has been made and a clearer picture of Fe-S cluster biogenesis is emerging, many questions remains unanswered. We believe that, in the coming years, NMR will be instrumental in increasing our understanding of mechanisms of eukaryotic Fe-S cluster biogenesis, how the defects of this process lead to human disease, and, finally, how disease states can be controlled. New breakthroughs in NMR techniques such as in-cell NMR [244,245,246,247,248,249], which make it possible to study protein structure and dynamics directly in cells, may also contribute to our future understanding of Fe-S cluster assembly and maturation.

## Figures and Tables

**Table 1:** Proteins involved in mitochondrial ISC machinery and related diseases.

Human Protein	UNIPROT ID	Yeast Ortholog	Diseases	Cofactors	Putative Functions
FXN	Q16595	Yfh1	Friedreich’s ataxia (FRDA) [64,65]	Fe^2+^	Proximal iron donor, controls iron entry and sulfur transfer to ISCU
ISCU	Q9H1K1	Isu1 and Isu2	ISCU myopathy [66,67,68]	[2Fe-2S]	Scaffold for Fe-S cluster assembly
FDX1	P10109			[2Fe-2S]	Electron donor in cluster assembly
FDX2 (FDX1L)	Q6P4F2	Yah1	Myopathy [69]	[2Fe-2S]	Electron donor in cluster assembly
NFS1	Q8WV90	Nfs1	Infantile mitochondrial complex II/III deficiency [70]	PLP	Cysteine desulfurase
ISD11 (LYRM4)	Q9HD34	Isd11	Respiratory chain deficiency [71]		Stabilization of NFS1 in cluster assembly
ACP (NDUFAB1)	O14561	Acp1		4′-phospho-pantetheine	Stabilization of NFS1-ISD11 complex in cluster assembly
GLRX5	Q86SX6	Grx5	Microcytic anemia and sideroblastic anemia [72,73]	[2Fe-2S]	Fe-S cluster carrier protein
ABCB7	O75027	Atm1	Sideroblastic anemia and ataxia [74]		Mitochondrial export
Mitoferrin1 (MFRN1)	Q9NYZ2	Mrs3 and Mrs4	Variant erythropoietic protoporphyria [75]		Mitochondrial iron importer
NUBPL	Q8TB37	Ind1	Mitochondrial encephalomyopathy [76]	[4Fe-4S]	Involved in Fe-S cluster transfer to Complex I
NFU1	Q9UMS0	Nfu1	Multiple mitochondrial dysfunction syndrome 1 (MMDS1) [47,48]	[4Fe-4S]	Fe-S delivery to specific recipients
ISCA1	Q9BUE6	Isa1	Multiple mitochondrial dysfunction syndrome 5 (MMDS5) [77,78]	[2Fe-2S], [4Fe-4S]	[4Fe-4S] cluster assembly
ISCA2	Q86U28	Isa2	Multiple mitochondrial dysfunction syndrome 4 (MMDS4) [79]	[2Fe-2S], [4Fe-4S]	[4Fe-4S] cluster assembly
BOLA1	Q9Y3E2	Bol1			Iron sensing/[2Fe-2S] delivery
BOLA3	Q53S33	Aim1	Multiple mitochondrial dysfunction syndrome 2 (MMDS2) [48]		Fe-S delivery to specific recipients
IBA57	Q5T440	Iba57	Multiple mitochondrial dysfunction syndrome 3 (MMDS3) [80,81]		[4Fe-4S] cluster assembly
HSC20	Q8IWL3	Jac1			J-type co-chaperone
HSC70	P38646	Ssq1	Parkinson’s disease [82]	ATP, ADP	Hsp70-type chaperone involved in cluster delivery

**Table 2:** Proteins involved in CIA machinery.

Human Protein	UNIPROT ID	Yeast Ortholog	Cofactors	Putative Functions
NUBP1	P53384	Nbp35	[4Fe-4S]	Scaffold protein for formation of a [4Fe-4S] cluster [211,212]
NUBP2	Q9Y5Y2	Cfd1	[4Fe-4S]	Scaffold protein for formation of a [4Fe-4S] cluster [211,212]
NDOR1	Q9UHB4	Tah18	FAD, FMN, NADPH	Electron transfer [224,225]
Anamorsin (CIAPIN1)	Q6FI81	Dre2	[2Fe-2S], [4Fe-4S]	Electron transfer [224,225]
GLRX3	O76003	Grx3 and Grx4	[2Fe-2S], GSH	Fe-S cluster transfer, iron trafficking [206,226,227]
BOLA2	Q9H3K6	Isd11	[4Fe-4S]	Fe-S cluster transfer [226,228]
NARFL (IOP1)	Q9H6Q4	Nar1		Adaptor protein to connect early and late-acting CIA components [216,229]
CIAO1	O76071	Cia1		CIA targeting complex [214]
MMS19	Q96T76	Met18		CIA targeting complex [217,222]
CIA2A (FAM96A)	Q9H5X1			Specific maturation factor of IRP1 [215]
CIA2B (FAM96B)	Q9Y3D0	Cia2		CIA targeting complex [215]
YAE1D1	Q9NRH1	Yae1		Specific maturation factor of the cytosolic ABCE1 [221]
ORAOV1	Q8WV07	YNL260C		Specific maturation factor of the cytosolic ABCE1 [221]

## References

[1] Waldron K.J., Rutherford J.C., Ford D., Robinson N.J. (2009). Metalloproteins and metal sensing. Nature.

[2] Finney L.A., O′Halloran T.V. (2003). Transition metal speciation in the cell: Insights from the chemistry of metal ion receptors. Science.

[3] Beinert H., Holm R.H., Munck E. (1997). Iron-sulfur clusters: Nature’s modular, multipurpose structures. Science.

[4] Frazzon J., Dean D.R. (2003). Formation of iron-sulfur clusters in bacteria: An emerging field in bioinorganic chemistry. Curr. Opin. Chem. Biol..

[5] Johnson D.C., Dean D.R., Smith A.D., Johnson M.K. (2005). Structure, function, and formation of biological iron-sulfur clusters. Annu. Rev. Biochem..

[6] Lill R. (2009). Function and biogenesis of iron-sulphur proteins. Nature.

[7] Py B., Barras F. (2010). Building Fe-S proteins: Bacterial strategies. Nat. Rev. Microbiol..

[8] Beinert H. (2000). Iron-sulfur proteins: Ancient structures, still full of surprises. J. Biol. Inorg. Chem..

[9] Lill R., Dutkiewicz R., Elsasser H.P., Hausmann A., Netz D.J.A., Pierik A.J., Stehling O., Urzica E., Muhlenhoff U. (2006). Mechanisms of iron-sulfur protein maturation in mitochondria, cytosol and nucleus of eukaryotes. Biochim. Biophys. Acta.

[10] Ayala-Castro C., Saini A., Outten F.W. (2008). Fe-S cluster assembly pathways in bacteria. Microbiol. Mol. Biol. Rev..

[11] Liu J., Chakraborty S., Hosseinzadeh P., Yu Y., Tian S., Petrik I., Bhagi A., Lu Y. (2014). Metalloproteins containing cytochrome, iron-sulfur, or copper redox centers. Chem. Rev..

[12] Flint D.H., Allen R.M. (1996). Iron-sulfur proteins with nonredox functions. Chem. Rev..

[13] Rouault T.A. (2006). The role of iron regulatory proteins in mammalian iron homeostasis and disease. Nat. Chem. Biol..

[14] Volz K. (2008). The functional duality of iron regulatory protein 1. Curr. Opin. Struct. Biol..

[15] Fleischhacker A.S., Kiley P.J. (2011). Iron-containing transcription factors and their roles as sensors. Curr. Opin. Chem. Biol..

[16] Tong W.H., Rouault T.A. (2007). Metabolic regulation of citrate and iron by aconitases: Role of iron-sulfur cluster biogenesis. Biometals.

[17] White M.F., Dillingham M.S. (2012). Iron-sulphur clusters in nucleic acid processing enzymes. Curr. Opin. Struct. Biol..

[18] O’Brien E., Holt M.E., Thompson M.K., Salay L.E., Ehlinger A.C., Chazin W.J., Barton J.K. (2017). The [4Fe4S] cluster of human DNA primase functions as a redox switch using DNA charge transport. Science.

[19] Veatch J.R., McMurray M.A., Nelson Z.W., Gottschling D.E. (2009). Mitochondrial dysfunction leads to nuclear genome instability via an iron-sulfur cluster defect. Cell.

[20] Netz D.J.A., Stith C.M., Stumpfig M., Kopf G., Vogel D., Genau H.M., Stodola J.L., Lill R., Burgers P.M.J., Pierik A.J. (2012). Eukaryotic DNA polymerases require an iron-sulfur cluster for the formation of active complexes. Nat. Chem. Biol..

[21] Rudolf J., Makrantoni V., Ingledew W.J., Stark M.J.R., White M.F. (2006). The DNA repair helicases XPD and FancJ have essential iron-sulfur domains. Mol. Cell.

[22] Schnackerz K.D., Dobritzsch D., Lindqvist Y., Cook P.F. (2004). Dihydropyrimidine dehydrogenase: A flavoprotein with four iron-sulfur clusters. Biochim. Biophys. Acta.

[23] Kispal G., Sipos K., Lange H., Fekete Z., Bedekovics T., Janaky T., Bassler J., Aguilar Netz D.J., Balk J., Rotte C. (2005). Biogenesis of cytosolic ribosomes requires the essential iron-sulphur protein Rli1p and mitochondria. EMBO J..

[24] Maio N., Rouault T.A. (2015). Iron-sulfur cluster biogenesis in mammalian cells: New insights into the molecular mechanisms of cluster delivery. Biochim. Biophys. Acta.

[25] Ollagnier-De Choudens S., Sanakis Y., Hewitson K.S., Roach P., Baldwin J.E., Munck E., Fontecave M. (2000). Iron-sulfur center of biotin synthase and lipoate synthase. Biochemistry.

[26] Lanz N.D., Booker S.J. (2015). Auxiliary iron-sulfur cofactors in radical SAM enzymes. Biochim. Biophys. Acta.

[27] Landgraf B.J., McCarthy E.L., Booker S.J. (2016). Radical S-Adenosylmethionine Enzymes in Human Health and Disease. Annu. Rev. Biochem..

[28] Qin S., Yin H., Yang C., Dou Y., Liu Z., Zhang P., Yu H., Huang Y., Feng J., Hao J. (2015). A magnetic protein biocompass. Nat. Mater..

[29] Roche B., Aussel L., Ezraty B., Mandin P., Py B., Barras F. (2013). Iron/sulfur proteins biogenesis in prokaryotes: Formation, regulation and diversity. Biochim. Biophys. Acta.

[30] Blanc B., Gerez C., Ollagnier de Choudens S. (2015). Assembly of Fe/S proteins in bacterial systems: Biochemistry of the bacterial ISC system. Biochim. Biophys. Acta.

[31] Prischi F., Konarev P.V., Iannuzzi C., Pastore C., Adinolfi S., Martin S.R., Svergun D.I., Pastore A. (2010). Structural bases for the interaction of frataxin with the central components of iron-sulphur cluster assembly. Nat. Commun..

[32] Adinolfi S., Iannuzzi C., Prischi F., Pastore C., Iametti S., Martin S.R., Bonomi F., Pastore A. (2009). Bacterial frataxin CyaY is the gatekeeper of iron-sulfur cluster formation catalyzed by IscS. Nat. Struct. Mol. Biol..

[33] Kim J.H., Bothe J.R., Alderson T.R., Markley J.L. (2015). Tangled web of interactions among proteins involved in iron-sulfur cluster assembly as unraveled by NMR, SAXS, chemical crosslinking, and functional studies. Biochim. Biophys. Acta.

[34] Gray M.W. (2012). Mitochondrial evolution. Cold Spring Harb. Perspect. Biol..

[35] Lill R., Muhlenhoff U. (2005). Iron-sulfur-protein biogenesis in eukaryotes. Trends Biochem. Sci..

[36] Stehling O., Lill R. (2013). The role of mitochondria in cellular iron-sulfur protein biogenesis: Mechanisms, connected processes, and diseases. Cold Spring Harb. Perspect. Biol..

[37] Braymer J.J., Lill R. (2017). Iron-sulfur cluster biogenesis and trafficking in mitochondria. J. Biol. Chem..

[38] Uzarska M.A., Dutkiewicz R., Freibert S.A., Lill R., Muehlenhoff U. (2013). The mitochondrial Hsp70 chaperone Ssq1 facilitates Fe/S cluster transfer from Isu1 to Grx5 by complex formation. Mol. Biol. Cell.

[39] Rodriguez-Manzaneque M.T., Tamarit J., Belli G., Ros J., Herrero E. (2002). Grx5 is a mitochondrial glutaredoxin required for the activity of iron/sulfur enzymes. Mol. Biol. Cell.

[40] Muhlenhoff U., Hoffmann B., Richter N., Rietzschel N., Spantgar F., Stehling O., Uzarska M.A., Lill R. (2015). Compartmentalization of iron between mitochondria and the cytosol and its regulation. Eur. J. Cell Biol..

[41] Lill R., Dutkiewicz R., Freibert S.A., Heidenreich T., Mascarenhas J., Netz D.J., Paul V.D., Pierik A.J., Richter N., Stumpfig M. (2015). The role of mitochondria and the CIA machinery in the maturation of cytosolic and nuclear iron-sulfur proteins. Eur. J. Cell Biol..

[42] Netz D.J., Mascarenhas J., Stehling O., Pierik A.J., Lill R. (2014). Maturation of cytosolic and nuclear iron-sulfur proteins. Trends Cell Biol..

[43] Beilschmidt L.K., Ollagnier de Choudens S., Fournier M., Sanakis I., Hograindleur M.A., Clemancey M., Blondin G., Schmucker S., Eisenmann A., Weiss A. (2017). ISCA1 is essential for mitochondrial Fe4S4 biogenesis in vivo. Nat. Commun..

[44] Sheftel A.D., Wilbrecht C., Stehling O., Niggemeyer B., Elsasser H.P., Muhlenhoff U., Lill R. (2012). The human mitochondrial ISCA1, ISCA2, and IBA57 proteins are required for [4Fe-4S] protein maturation. Mol. Biol. Cell.

[45] Muhlenhoff U., Richter N., Pines O., Pierik A.J., Lill R. (2011). Specialized function of yeast Isa1 and Isa2 proteins in the maturation of mitochondrial [4Fe-4S] proteins. J. Biol. Chem..

[46] Melber A., Na U., Vashisht A., Weiler B.D., Lill R., Wohlschlegel J.A., Winge D.R. (2016). Role of Nfu1 and Bol3 in iron-sulfur cluster transfer to mitochondrial clients. Elife.

[47] Navarro-Sastre A., Tort F., Stehling O., Uzarska M.A., Arranz J.A., Del Toro M., Labayru M.T., Landa J., Font A., Garcia-Villoria J. (2011). A fatal mitochondrial disease is associated with defective NFU1 function in the maturation of a subset of mitochondrial Fe-S proteins. Am. J. Hum. Genet..

[48] Cameron J.M., Janer A., Levandovskiy V., Mackay N., Rouault T.A., Tong W.H., Ogilvie I., Shoubridge E.A., Robinson B.H. (2011). Mutations in iron-sulfur cluster scaffold genes NFU1 and BOLA3 cause a fatal deficiency of multiple respiratory chain and 2-oxoacid dehydrogenase enzymes. Am. J. Hum. Genet..

[49] Rouault T.A. (2012). Biogenesis of iron-sulfur clusters in mammalian cells: New insights and relevance to human disease. Dis. Model. Mech..

[50] Stehling O., Wilbrecht C., Lill R. (2014). Mitochondrial iron-sulfur protein biogenesis and human disease. Biochimie.

[51] Ye H., Rouault T.A. (2010). Human iron-sulfur cluster assembly, cellular iron homeostasis, and disease. Biochemistry.

[52] Cheng H., Markley J.L. (1995). NMR Spectroscopic Studies of Paramagnetic Proteins—Iron-Sulfur Proteins. Annu. Rev. Biophys. Biomol. Struct..

[53] Bertini I., Couture M.M.J., Donaire A., Eltis L.D., Felli I.C., Luchinat C., Piccioli M., Rosato A. (1996). The solution structure refinement of the paramagnetic reduced high-potential iron-sulfur protein I from Ectothiorhodospira halophila by using stable isotope labeling and nuclear relaxation. Eur. J. Biochem..

[54] Xia B., Jenk D., LeMaster D.M., Westler W.M., Markley J.L. (2000). Electron-nuclear interactions in two prototypical [2Fe-2S] proteins: Selective (chiral) deuteration and analysis of H-1 and H-2 NMR signals from the α and β hydrogens of cysteinyl residues that ligate the iron in the active sites of human ferredoxin and Anabaena 7120 vegetative ferredoxin. Arch. Biochem. Biophys..

[55] Cheng H., Westler W.M., Xia B., Oh B.H., Markley J.L. (1995). Protein expression, selective isotopic labeling, and analysis of hyperfine-shifted NMR signals of Anabaena 7120 vegetative [2Fe-2S] ferredoxin. Arch. Biochem. Biophys..

[56] Bertini I., Donaire A., Luchinat C., Rosato A. (1997). Paramagnetic relaxation as a tool for solution structure determination: Clostridium pasteurianum ferredoxin as an example. Proteins-Struct. Funct. Genet..

[57] Machonkin T.E., Westler W.M., Markley J.L. (2002). ^13^C{^13^C} 2D NMR: A novel strategy for the study of paramagnetic proteins with slow electronic relaxation rates. J. Am. Chem. Soc..

[58] Bermel W., Bertini I., Felli I.C., Kummerle R., Pierattelli R. (2006). Novel ^13^C direct detection experiments, including extension to the third dimension, to perform the complete assignment of proteins. J. Magn. Reson..

[59] Banci L., Camponeschi F., Ciofi-Baffoni S., Piccioli M. (2018). The NMR contribution to protein-protein networking in Fe-S protein maturation. J. Biol. Inorg. Chem..

[60] Ciofi-Baffoni S., Gallo A., Muzzioli R., Piccioli M. (2014). The IR-^15^N-HSQC-AP experiment: A new tool for NMR spectroscopy of paramagnetic molecules. J. Biomol. NMR.

[61] Lian L.Y. (2013). NMR studies of weak protein-protein interactions. Prog. Nucl. Magn. Reson. Spectrosc..

[62] Piccioli M., Turano P. (2015). Transient iron coordination sites in proteins: Exploiting the dual nature of paramagnetic NMR. Coord. Chem. Rev..

[63] Ciofi-Baffoni S., Nasta V., Banci L. (2017). Protein networks in the maturation of human iron-sulfur proteins. Metallomics.

[64] Campuzano V., Montermini L., Molto M.D., Pianese L., Cossee M., Cavalcanti F., Monros E., Rodius F., Duclos F., Monticelli A. (1996). Friedreich’s ataxia: Autosomal recessive disease caused by an intronic GAA triplet repeat expansion. Science.

[65] Santos R., Lefevre S., Sliwa D., Seguin A., Camadro J.M., Lesuisse E. (2010). Friedreich ataxia: Molecular mechanisms, redox considerations, and therapeutic opportunities. Antioxid. Redox Signal..

[66] Crooks D.R., Jeong S.Y., Tong W.H., Ghosh M.C., Olivierre H., Haller R.G., Rouault T.A. (2012). Tissue specificity of a human mitochondrial disease: Differentiation-enhanced mis-splicing of the Fe-S scaffold gene ISCU renders patient cells more sensitive to oxidative stress in ISCU myopathy. J. Biol. Chem..

[67] Olsson A., Lind L., Thornell L.E., Holmberg M. (2008). Myopathy with lactic acidosis is linked to chromosome 12q23.3-24.11 and caused by an intron mutation in the ISCU gene resulting in a splicing defect. Hum. Mol. Genet..

[68] Mochel F., Knight M.A., Tong W.H., Hernandez D., Ayyad K., Taivassalo T., Andersen P.M., Singleton A., Rouault T.A., Fischbeck K.H. (2008). Splice mutation in the iron-sulfur cluster scaffold protein ISCU causes myopathy with exercise intolerance. Am. J. Hum. Genet..

[69] Spiegel R., Saada A., Halvardson J., Soiferman D., Shaag A., Edvardson S., Horovitz Y., Khayat M., Shalev S.A., Feuk L. (2014). Deleterious mutation in FDX1L gene is associated with a novel mitochondrial muscle myopathy. Eur. J. Hum. Genet..

[70] Farhan S.M., Wang J., Robinson J.F., Lahiry P., Siu V.M., Prasad C., Kronick J.B., Ramsay D.A., Rupar C.A., Hegele R.A. (2014). Exome sequencing identifies NFS1 deficiency in a novel Fe-S cluster disease, infantile mitochondrial complex II/III deficiency. Mol. Genet. Genom. Med..

[71] Invernizzi F., Tigano M., Dallabona C., Donnini C., Ferrero I., Cremonte M., Ghezzi D., Lamperti C., Zeviani M. (2013). A homozygous mutation in LYRM7/MZM1L associated with early onset encephalopathy, lactic acidosis, and severe reduction of mitochondrial complex III activity. Hum. Mutat..

[72] Ye H., Jeong S.Y., Ghosh M.C., Kovtunovych G., Silvestri L., Ortillo D., Uchida N., Tisdale J., Camaschella C., Rouault T.A. (2010). Glutaredoxin 5 deficiency causes sideroblastic anemia by specifically impairing heme biosynthesis and depleting cytosolic iron in human erythroblasts. J. Clin. Investig..

[73] Wingert R.A., Galloway J.L., Barut B., Foott H., Fraenkel P., Axe J.L., Weber G.J., Dooley K., Davidson A.J., Schmid B. (2005). Deficiency of glutaredoxin 5 reveals Fe-S clusters are required for vertebrate haem synthesis. Nature.

[74] Allikmets R., Raskind W.H., Hutchinson A., Schueck N.D., Dean M., Koeller D.M. (1999). Mutation of a putative mitochondrial iron transporter gene (ABC7) in X-linked sideroblastic anemia and ataxia (XLSA/A). Hum. Mol. Genet..

[75] Shaw G.C., Cope J.J., Li L., Corson K., Hersey C., Ackermann G.E., Gwynn B., Lambert A.J., Wingert R.A., Traver D. (2006). Mitoferrin is essential for erythroid iron assimilation. Nature.

[76] Calvo S.E., Tucker E.J., Compton A.G., Kirby D.M., Crawford G., Burtt N.P., Rivas M., Guiducci C., Bruno D.L., Goldberger O.A. (2010). High-throughput, pooled sequencing identifies mutations in NUBPL and FOXRED1 in human complex I deficiency. Nat. Genet..

[77] Torraco A., Stehling O., Stumpfig C., Rosser R., De Rasmo D., Fiermonte G., Verrigni D., Rizza T., Vozza A., Di Nottia M. (2018). ISCA1 Mutation in a Patient with Infantile-Onset Leukodystrophy Causes Defects in Mitochondrial [4Fe-4S] Proteins. Hum. Mol. Genet..

[78] Shukla A., Hebbar M., Srivastava A., Kadavigere R., Upadhyai P., Kanthi A., Brandau O., Bielas S., Girisha K.M. (2017). Homozygous p.(Glu87Lys) variant in ISCA1 is associated with a multiple mitochondrial dysfunctions syndrome. J. Hum. Genet..

[79] Al-Hassnan Z.N., Al-Dosary M., Alfadhel M., Faqeih E.A., Alsagob M., Kenana R., Almass R., Al-Harazi O.S., Al-Hindi H., Malibari O.I. (2015). ISCA2 mutation causes infantile neurodegenerative mitochondrial disorder. J. Med. Genet..

[80] Torraco A., Ardissone A., Invernizzi F., Rizza T., Fiermonte G., Niceta M., Zanetti N., Martinelli D., Vozza A., Verrigni D. (2017). Novel mutations in IBA57 are associated with leukodystrophy and variable clinical phenotypes. J. Neurol..

[81] Lossos A., Stumpfig C., Stevanin G., Gaussen M., Zimmerman B., Mundwiller E., Asulin M., Chamma L., Sheffer R., Misk A. (2015). Fe/S protein assembly gene IBA57 mutation causes hereditary spastic paraplegia. Neurology.

[82] De Mena L., Coto E., Sanchez-Ferrero E., Ribacoba R., Guisasola L.M., Salvador C., Blazquez M., Alvarez V. (2009). Mutational screening of the mortalin gene (HSPA9) in Parkinson’s disease. J. Neural. Transm. (Vienna).

[83] Markley J.L., Kim J.H., Dai Z., Bothe J.R., Cai K., Frederick R.O., Tonelli M. (2013). Metamorphic protein IscU alternates conformations in the course of its role as the scaffold protein for iron-sulfur cluster biosynthesis and delivery. FEBS Lett..

[84] Cai K., Frederick R.O., Kim J.H., Reinen N.M., Tonelli M., Markley J.L. (2013). Human mitochondrial chaperone (mtHSP70) and cysteine desulfurase (NFS1) bind preferentially to the disordered conformation, whereas co-chaperone (HSC20) binds to the structured conformation of the iron-sulfur cluster scaffold protein (ISCU). J. Biol. Chem..

[85] Kim J.H., Tonelli M., Markley J.L. (2012). Disordered form of the scaffold protein IscU is the substrate for iron-sulfur cluster assembly on cysteine desulfurase. Proc. Nat. Acad. Sci. USA.

[86] Bothe J.R., Tonelli M., Ali I.K., Dai Z., Frederick R.O., Westler W.M., Markley J.L. (2015). The Complex Energy Landscape of the Protein IscU. Biophys. J..

[87] Kim J.H., Tonelli M., Kim T., Markley J.L. (2012). Three-Dimensional Structure and Determinants of Stability of the Iron-Sulfur Cluster Scaffold Protein IscU from *Escherichia coli*. Biochemistry.

[88] Ramelot T.A., Cort J.R., Goldsmith-Fischman S., Kornhaber G.J., Xiao R., Shastry R., Acton T.B., Honig B., Montelione G.T., Kennedy M.A. (2004). Solution NMR structure of the iron-sulfur cluster assembly protein U (IscU) with zinc bound at the active site. J. Mol. Biol..

[89] Iannuzzi C., Adrover M., Puglisi R., Yan R., Temussi P.A., Pastore A. (2014). The role of zinc in the stability of the marginally stable IscU scaffold protein. Protein Sci..

[90] Tsai C.L., Barondeau D.P. (2010). Human Frataxin Is an Allosteric Switch That Activates the Fe-S Cluster Biosynthetic Complex. Biochemistry.

[91] Fox N.G., Martelli A., Nabhan J.F., Janz J., Borkowska O., Bulawa C., Yue W.W. (2018). Zinc(II) binding on human wild-type ISCU and Met140 variants modulates NFS1 desulfurase activity. Biochimie.

[92] Boniecki M.T., Freibert S.A., Muhlenhoff U., Lill R., Cygler M. (2017). Structure and functional dynamics of the mitochondrial Fe/S cluster synthesis complex. Nat. Commun..

[93] Sensi S.L., Ton-That D., Sullivan P.G., Jonas E.A., Gee K.R., Kaczmarek L.K., Weiss J.H. (2003). Modulation of mitochondrial function by endogenous Zn^2+^ pools. Proc. Nat. Acad. Sci. USA.

[94] Brown A.M., Kristal B.S., Effron M.S., Shestopalov A.I., Ullucci P.A., Sheu K.F., Blass J.P., Cooper A.J. (2000). Zn^2+^ inhibits α-ketoglutarate-stimulated mitochondrial respiration and the isolated α-ketoglutarate dehydrogenase complex. J. Biol. Chem..

[95] Dai Z., Tonelli M., Markley J.L. (2012). Metamorphic protein IscU changes conformation by *cis*-*trans* isomerizations of two peptidyl-prolyl peptide bonds. Biochemistry.

[96] Kim J.H., Tonelli M., Frederick R.O., Chow D.C., Markley J.L. (2012). Specialized Hsp70 chaperone (HscA) binds preferentially to the disordered form, whereas J-protein (HscB) binds preferentially to the structured form of the iron-sulfur cluster scaffold protein (IscU). J. Biol. Chem..

[97] Yan R., Kelly G., Pastore A. (2014). The scaffold protein IscU retains a structured conformation in the Fe-S cluster assembly complex. ChemBioChem.

[98] Muhlenhoff U., Balk J., Richhardt N., Kaiser J.T., Sipos K., Kispal G., Lill R. (2004). Functional characterization of the eukaryotic cysteine desulfurase Nfs1p from Saccharomyces cerevisiae. J. Biol. Chem..

[99] Rocha A.G., Knight S.A.B., Pandey A., Yoon H., Pain J., Pain D., Dancis A. (2018). Cysteine desulfurase is regulated by phosphorylation of Nfs1 in yeast mitochondria. Mitochondrion.

[100] Adam A.C., BornhÖvd C., Prokisch H., Neupert W., Hell K. (2006). The Nfs1 interacting protein Isd11 has an essential role in Fe/S cluster biogenesis in mitochondria. EMBO J..

[101] Shi Y., Ghosh M.C., Tong W.H., Rouault T.A. (2009). Human ISD11 is essential for both iron-sulfur cluster assembly and maintenance of normal cellular iron homeostasis. Hum. Mol. Genet..

[102] Wiedemann N., Urzica E., Guiard B., Muller H., Lohaus C., Meyer H.E., Ryan M.T., Meisinger C., Muhlenhoff U., Lill R. (2006). Essential role of Isd11 in mitochondrial iron-sulfur cluster synthesis on Isu scaffold proteins. EMBO J..

[103] Angerer H. (2013). The superfamily of mitochondrial Complex1_LYR motif-containing (LYRM) proteins. Biochem. Soc. Trans..

[104] Angerer H. (2015). Eukaryotic LYR Proteins Interact with Mitochondrial Protein Complexes. Biology.

[105] Kastaniotis A.J., Autio K.J., Keratar J.M., Monteuuis G., Makela A.M., Nair R.R., Pietikainen L.P., Shvetsova A., Chen Z., Hiltunen J.K. (2017). Mitochondrial fatty acid synthesis, fatty acids and mitochondrial physiology. Biochim. Biophys. Acta.

[106] Hiltunen J.K., Autio K.J., Schonauer M.S., Kursu V.A., Dieckmann C.L., Kastaniotis A.J. (2010). Mitochondrial fatty acid synthesis and respiration. Biochim. Biophys. Acta.

[107] Byers D.M., Gong H. (2007). Acyl carrier protein: Structure-function relationships in a conserved multifunctional protein family. Biochem. Cell Biol..

[108] Wu B.N., Zhang Y.M., Rock C.O., Zheng J.J. (2009). Structural modification of acyl carrier protein by butyryl group. Protein Sci..

[109] Zornetzer G.A., White R.D., Markley J.L., Fox B.G. (2006). Preparation of isotopically labeled spinach acyl-acyl carrier protein for NMR structural studies. Protein Expr. Purif..

[110] Findlow S.C., Winsor C., Simpson T.J., Crosby J., Crump M.P. (2003). Solution structure and dynamics of oxytetracycline polyketide synthase acyl carrier protein from Streptomyces rimosus. Biochemistry.

[111] Xu G.Y., Tam A., Lin L., Hixon J., Fritz C.C., Powers R. (2001). Solution structure of *B. subtilis* acyl carrier protein. Structure.

[112] Mayo K.H., Prestegard J.H. (1985). Acyl carrier protein from *Escherichia coli* Structural characterization of short-chain acylated acyl carrier proteins by NMR. Biochemistry.

[113] Nguyen C., Haushalter R.W., Lee D.J., Markwick P.R., Bruegger J., Caldara-Festin G., Finzel K., Jackson D.R., Ishikawa F., O’Dowd B. (2014). Trapping the dynamic acyl carrier protein in fatty acid biosynthesis. Nature.

[114] Cronan J.E. (2014). The chain-flipping mechanism of ACP (acyl carrier protein)-dependent enzymes appears universal. Biochem. J..

[115] Beld J., Cang H., Burkart M.D. (2014). Visualizing the chain-flipping mechanism in fatty-acid biosynthesis. Angew. Chem..

[116] Herrera M.G., Pignataro M.F., Noguera M.E., Cruz K.M., Santos J. (2018). Rescuing the Rescuer: On the Protein Complex between the Human Mitochondrial Acyl Carrier Protein and ISD11. ACS Chem. Biol..

[117] Cai K., Frederick R.O., Tonelli M., Markley J.L. (2017). Mitochondrial cysteine desulfurase and ISD11 coexpressed in *Escherichia coli* yield complex containing acyl carrier protein. ACS Chem. Biol..

[118] Yan R., Friemel M., Aloisi C., Huynen M., Taylor I.A., Leimkuhler S., Pastore A. (2016). The Eukaryotic-Specific ISD11 Is a Complex-Orphan Protein with Ability to Bind the Prokaryotic IscS. PLoS ONE.

[119] Kim Y., Kovrigin E.L., Eletr Z. (2006). NMR studies of Escherichia coli acyl carrier protein: Dynamic and structural differences of the apo- and holo-forms. Biochem. Biophys. Res. Commun..

[120] Gully D., Moinier D., Loiseau L., Bouveret E. (2003). New partners of acyl carrier protein detected in *Escherichia coli* by tandem affinity purification. FEBS Lett..

[121] Marinoni E.N., de Oliveira J.S., Nicolet Y., Raulfs E.C., Amara P., Dean D.R., Fontecilla-Camps J.C. (2012). (IscS-IscU)_2_ complex structures provide insights into Fe_2_S_2_ biogenesis and transfer. Angew. Chem. Int. Ed. Engl..

[122] Shi R., Proteau A., Villarroya M., Moukadiri I., Zhang L., Trempe J.F., Matte A., Armengod M.E., Cygler M. (2010). Structural basis for Fe-S cluster assembly and tRNA thiolation mediated by IscS protein-protein interactions. PLoS Biol..

[123] Cupp-Vickery J.R., Urbina H., Vickery L.E. (2003). Crystal structure of IscS, a cysteine desulfurase from *Escherichia coli*. J. Mol. Biol..

[124] Van Vranken J.G., Jeong M.Y., Wei P., Chen Y.C., Gygi S.P., Winge D.R., Rutter J. (2016). The mitochondrial acyl carrier protein (ACP) coordinates mitochondrial fatty acid synthesis with iron sulfur cluster biogenesis. Elife.

[125] Cory S.A., Van Vranken J.G., Brignole E.J., Patra S., Winge D.R., Drennan C.L., Rutter J., Barondeau D.P. (2017). Structure of human Fe-S assembly subcomplex reveals unexpected cysteine desulfurase architecture and acyl-ACP-ISD11 interactions. Proc. Nat. Acad. Sci. USA.

[126] Zhu J., Vinothkumar K.R., Hirst J. (2016). Structure of mammalian respiratory complex I. Nature.

[127] Fiedorczuk K., Letts J.A., Degliesposti G., Kaszuba K., Skehel M., Sazanov L.A. (2016). Atomic structure of the entire mammalian mitochondrial complex I. Nature.

[128] Brown A., Rathore S., Kimanius D., Aibara S., Bai X.C., Rorbach J., Amunts A., Ramakrishnan V. (2017). Structures of the human mitochondrial ribosome in native states of assembly. Nat. Struct. Mol. Biol..

[129] Cai K., Frederick R.O., Dashti H., Markley J.L. (2018). Architectural Features of Human Mitochondrial Cysteine Desulfurase Complexes from Crosslinking Mass Spectrometry and Small Angle X-ray Scattering. Structure.

[130] Muhlenhoff U., Richhardt N., Gerber J., Lill R. (2002). Characterization of iron-sulfur protein assembly in isolated mitochondria: A requirement for ATP, NADH, and reduced iron. J. Biol. Chem..

[131] Muhlenhoff U., Gerber J., Richhardt N., Lill R. (2003). Components involved in assembly and dislocation of iron-sulfur clusters on the scaffold protein Isu1p. EMBO J..

[132] Webert H., Freibert S.A., Gallo A., Heidenreich T., Linne U., Amlacher S., Hurt E., Muhlenhoff U., Banci L., Lill R. (2014). Functional reconstitution of mitochondrial Fe/S cluster synthesis on Isu1 reveals the involvement of ferredoxin. Nat. Commun..

[133] Yan R., Adinolfi S., Pastore A. (2015). Ferredoxin, in conjunction with NADPH and ferredoxin-NADP reductase, transfers electrons to the IscS/IscU complex to promote iron-sulfur cluster assembly. Biochim. Biophys. Acta.

[134] Ewen K.M., Ringle M., Bernhardt R. (2012). Adrenodoxin—A versatile ferredoxin. IUBMB Life.

[135] Sheftel A.D., Stehling O., Pierik A.J., Elsasser H.P., Muhlenhoff U., Webert H., Hobler A., Hannemann F., Bernhardt R., Lill R. (2010). Humans possess two mitochondrial ferredoxins, Fdx1 and Fdx2, with distinct roles in steroidogenesis, heme, and Fe/S cluster biosynthesis. Proc. Natl. Acad. Sci. USA.

[136] Shi Y., Ghosh M., Kovtunovych G., Crooks D.R., Rouault T.A. (2012). Both human ferredoxins 1 and 2 and ferredoxin reductase are important for iron-sulfur cluster biogenesis. Biochim. Biophys. Acta.

[137] Cai K., Tonelli M., Frederick R.O., Markley J.L. (2017). Human mitochondrial ferredoxin 1 (FDX1) and ferredoxin 2 (FDX2) both bind cysteine desulfurase and donate electrons for iron-sulfur cluster biosynthesis. Biochemistry.

[138] Kurisu G., Kusunoki M., Katoh E., Yamazaki T., Teshima K., Onda Y., Kimata-Ariga Y., Hase T. (2001). Structure of the electron transfer complex between ferredoxin and ferredoxin-NADP^+^ reductase. Nat. Struct. Biol..

[139] Yoon T., Cowan J.A. (2003). Iron-sulfur cluster biosynthesis. Characterization of frataxin as an iron donor for assembly of [2Fe-2S] clusters in ISU-type proteins. J. Am. Chem. Soc..

[140] Kondapalli K.C., Kok N.M., Dancis A., Stemmler T.L. (2008). Drosophila frataxin: An iron chaperone during cellular Fe-S cluster bioassembly. Biochemistry.

[141] Melber A., Winge D.R. (2018). Steps Toward Understanding Mitochondrial Fe/S Cluster Biogenesis. Methods Enzymol..

[142] Lindahl P.A., Moore M.J. (2016). Labile Low-Molecular-Mass Metal Complexes in Mitochondria: Trials and Tribulations of a Burgeoning Field. Biochemistry.

[143] Anzovino A., Lane D.J., Huang M.L., Richardson D.R. (2014). Fixing frataxin: ‘Ironing out’ the metabolic defect in Friedreich’s ataxia. Br. J. Pharmacol..

[144] Vaubel R.A., Isaya G. (2013). Iron-sulfur cluster synthesis, iron homeostasis and oxidative stress in Friedreich ataxia. Mol. Cell. Neurosci..

[145] Nair M., Adinolfi S., Pastore C., Kelly G., Temussi P., Pastore A. (2004). Solution structure of the bacterial frataxin ortholog, CyaY: Mapping the iron binding sites. Structure.

[146] He Y., Alam S.L., Proteasa S.V., Zhang Y., Lesuisse E., Dancis A., Stemmler T.L. (2004). Yeast frataxin solution structure, iron binding, and ferrochelatase interaction. Biochemistry.

[147] Musco G., Stier G., Kolmerer B., Adinolfi S., Martin S., Frenkiel T., Gibson T., Pastore A. (2000). Towards a structural understanding of Friedreich’s ataxia: The solution structure of frataxin. Structure.

[148] Parent A., Elduque X., Cornu D., Belot L., Le Caer J.P., Grandas A., Toledano M.B., D’Autreaux B. (2015). Mammalian frataxin directly enhances sulfur transfer of NFS1 persulfide to both ISCU and free thiols. Nat. Commun..

[149] Bridwell-Rabb J., Fox N.G., Tsai C.L., Winn A.M., Barondeau D.P. (2014). Human frataxin activates Fe-S cluster biosynthesis by facilitating sulfur transfer chemistry. Biochemistry.

[150] Bridwell-Rabb J., Iannuzzi C., Pastore A., Barondeau D.P. (2012). Effector role reversal during evolution: The case of frataxin in Fe-S cluster biosynthesis. Biochemistry.

[151] Cook J.D., Bencze K.Z., Jankovic A.D., Crater A.K., Busch C.N., Bradley P.B., Stemmler A.J., Spaller M.R., Stemmler T.L. (2006). Monomeric yeast frataxin is an iron-binding protein. Biochemistry.

[152] Yoon H., Knight S.A.B., Pandey A., Pain J., Turkarslan S., Pain D., Dancis A. (2015). Turning Saccharomyces cerevisiae into a Frataxin-Independent Organism. PLoS Genet..

[153] Yoon H., Knight S.A., Pandey A., Pain J., Zhang Y., Pain D., Dancis A. (2014). Frataxin-bypassing Isu1: Characterization of the bypass activity in cells and mitochondria. Biochem. J..

[154] Yoon H., Golla R., Lesuisse E., Pain J., Donald J.E., Lyver E.R., Pain D., Dancis A. (2012). Mutation in the Fe-S scaffold protein Isu bypasses frataxin deletion. Biochem. J..

[155] Colin F., Martelli A., Clemancey M., Latour J.M., Gambarelli S., Zeppieri L., Birck C., Page A., Puccio H., Ollagnier de Choudens S. (2013). Mammalian frataxin controls sulfur production and iron entry during *de novo* Fe_4_S_4_ cluster assembly. J. Am. Chem. Soc..

[156] Cai K., Frederick R.O., Tonelli M., Markley J.L. (2018). Interactions of iron-bound frataxin with ISCU and ferredoxin on the cysteine desulfurase complex leading to Fe-S cluster assembly. J. Inorg. Biochem..

[157] Cai K., Frederick R.O., Tonelli M., Markley J.L. (2018). ISCU(M108I) and ISCU(D39V) Differ from Wild-Type ISCU in Their Failure to Form Cysteine Desulfurase Complexes Containing Both Frataxin and Ferredoxin. Biochemistry.

[158] Yan R., Konarev P.V., Iannuzzi C., Adinolfi S., Roche B., Kelly G., Simon L., Martin S.R., Py B., Barras F. (2013). Ferredoxin competes with bacterial frataxin in binding to the desulfurase IscS. J. Biol. Chem..

[159] Kim J.H., Frederick R.O., Reinen N.M., Troupis A.T., Markley J.L. (2013). [2Fe-2S]-Ferredoxin binds directly to cysteine desulfurase and supplies an electron for iron-sulfur cluster assembly but is displaced by the scaffold protein or bacterial frataxin. J. Am. Chem. Soc..

[160] Pastore C., Adinolfi S., Huynen M.A., Rybin V., Martin S., Mayer M., Bukau B., Pastore A. (2006). YfhJ, a molecular adaptor in iron-sulfur cluster formation or a frataxin-like protein?. Structure.

[161] Kim J.H., Bothe J.R., Frederick R.O., Holder J.C., Markley J.L. (2014). Role of IscX in iron-sulfur cluster biogenesis in *Escherichia coli*. J. Am. Chem. Soc..

[162] Adinolfi S., Puglisi R., Crack J.C., Iannuzzi C., Dal Piaz F., Konarev P.V., Svergun D.I., Martin S., Le Brun N.E., Pastore A. (2018). The Molecular Bases of the Dual Regulation of Bacterial Iron Sulfur Cluster Biogenesis by CyaY and IscX. Front. Mol. Biosci..

[163] De Vries S.J., van Dijk M., Bonvin A.M. (2010). The HADDOCK web server for data-driven biomolecular docking. Nat. Protoc..

[164] Manicki M., Majewska J., Ciesielski S., Schilke B., Blenska A., Kominek J., Marszalek J., Craig E.A., Dutkiewicz R. (2014). Overlapping binding sites of the frataxin homologue assembly factor and the heat shock protein 70 transfer factor on the Isu iron-sulfur cluster scaffold protein. J. Biol. Chem..

[165] Kato S., Mihara H., Kurihara T., Takahashi Y., Tokumoto U., Yoshimura T., Esaki N. (2002). Cys-328 of IscS and Cys-63 of IscU are the sites of disulfide bridge formation in a covalently bound IscS/IscU complex: Implications for the mechanism of iron-sulfur cluster assembly. Proc. Natl. Acad. Sci. USA.

[166] Smith A.D., Frazzon J., Dean D.R., Johnson M.K. (2005). Role of conserved cysteines in mediating sulfur transfer from IscS to IscU. FEBS Lett..

[167] Vickery L.E., Cupp-Vickery J.R. (2007). Molecular chaperones HscA/Ssq1 and HscB/Jac1 and their roles in iron-sulfur protein maturation. Crit. Rev. Biochem. Mol. Biol..

[168] Rouault T.A. (2015). Mammalian iron-sulphur proteins: Novel insights into biogenesis and function. Nat. Rev. Mol. Cell Biol..

[169] Londono C., Osorio C., Gama V., Alzate O. (2012). Mortalin, apoptosis, and neurodegeneration. Biomolecules.

[170] Kaul S.C., Deocaris C.C., Wadhwa R. (2007). Three faces of mortalin: A housekeeper, guardian and killer. Exp. Gerontol..

[171] Craig E.A., Marszalek J. (2017). How Do J-Proteins Get Hsp70 to Do So Many Different Things?. Trends Biochem. Sci..

[172] Kampinga H.H., Craig E.A. (2010). The HSP70 chaperone machinery: J proteins as drivers of functional specificity. Nat. Rev. Mol. Cell Biol..

[173] Majewska J., Ciesielski S.J., Schilke B., Kominek J., Blenska A., Delewski W., Song J.Y., Marszalek J., Craig E.A., Dutkiewicz R. (2013). Binding of the chaperone Jac1 protein and cysteine desulfurase Nfs1 to the iron-sulfur cluster scaffold Isu protein is mutually exclusive. J. Biol. Chem..

[174] Maio N., Kim K.S., Singh A., Rouault T.A. (2017). A Single Adaptable Cochaperone-Scaffold Complex Delivers Nascent Iron-Sulfur Clusters to Mammalian Respiratory Chain Complexes I-III. Cell Metab..

[175] Maio N., Singh A., Uhrigshardt H., Saxena N., Tong W.H., Rouault T.A. (2014). Cochaperone binding to LYR motifs confers specificity of iron sulfur cluster delivery. Cell Metab..

[176] Hoff K.G., Cupp-Vickery J.R., Vickery L.E. (2003). Contributions of the LPPVK motif of the iron-sulfur template protein IscU to interactions with the Hsc66-Hsc20 chaperone system. J. Biol. Chem..

[177] Dutkiewicz R., Nowak M., Craig E.A., Marszalek J. (2017). Fe-S Cluster Hsp70 Chaperones: The ATPase Cycle and Protein Interactions. Methods Enzymol..

[178] Miao B.J., Davis J.E., Craig E.A. (1997). Mge1 functions as a nucleotide release factor for Ssc1, a mitochondrial Hsp70 of Saccharomyces cerevisiae. J. Mol. Biol..

[179] Shakamuri P., Zhang B., Johnson M.K. (2012). Monothiol glutaredoxins function in storing and transporting [Fe_2_S_2_] clusters assembled on IscU scaffold proteins. J. Am. Chem. Soc..

[180] Johansson C., Roos A.K., Montano S.J., Sengupta R., Filippakopoulos P., Guo K., von Delft F., Holmgren A., Oppermann U., Kavanagh K.L. (2011). The crystal structure of human GLRX5: Iron-sulfur cluster co-ordination, tetrameric assembly and monomer activity. Biochem. J..

[181] Banci L., Brancaccio D., Ciofi-Baffoni S., Del Conte R., Gadepalli R., Mikolajczyk M., Neri S., Piccioli M., Winkelmann J. (2014). [2Fe-2S] cluster transfer in iron-sulfur protein biogenesis. Proc. Natl. Acad. Sci. USA.

[182] Mapolelo D.T., Zhang B., Randeniya S., Albetel A.N., Li H., Couturier J., Outten C.E., Rouhier N., Johnson M.K. (2013). Monothiol glutaredoxins and A-type proteins: Partners in Fe-S cluster trafficking. Dalton Trans..

[183] Maio N., Rouault T.A. (2016). Mammalian Fe-S proteins: Definition of a consensus motif recognized by the co-chaperone HSC20. Metallomics.

[184] Agar J.N., Krebs C., Frazzon J., Huynh B.H., Dean D.R., Johnson M.K. (2000). IscU as a scaffold for iron-sulfur cluster biosynthesis: Sequential assembly of [2Fe-2S] and [4Fe-4S] clusters in IscU. Biochemistry.

[185] Smith A.D., Jameson G.N., Dos Santos P.C., Agar J.N., Naik S., Krebs C., Frazzon J., Dean D.R., Huynh B.H., Johnson M.K. (2005). NifS-mediated assembly of [4Fe-4S] clusters in the N- and C-terminal domains of the NifU scaffold protein. Biochemistry.

[186] Unciuleac M.C., Chandramouli K., Naik S., Mayer S., Huynh B.H., Johnson M.K., Dean D.R. (2007). In vitro activation of apo-aconitase using a [4Fe-4S] cluster-loaded form of the IscU [Fe-S] cluster scaffolding protein. Biochemistry.

[187] Ajit Bolar N., Vanlander A.V., Wilbrecht C., Van der Aa N., Smet J., De Paepe B., Vandeweyer G., Kooy F., Eyskens F., De Latter E. (2013). Mutation of the iron-sulfur cluster assembly gene IBA57 causes severe myopathy and encephalopathy. Hum. Mol. Genet..

[188] Brancaccio D., Gallo A., Mikolajczyk M., Zovo K., Palumaa P., Novellino E., Piccioli M., Ciofi-Baffoni S., Banci L. (2014). Formation of [4Fe-4S] clusters in the mitochondrial iron-sulfur cluster assembly machinery. J. Am. Chem. Soc..

[189] Brancaccio D., Gallo A., Piccioli M., Novellino E., Ciofi-Baffoni S., Banci L. (2017). [4Fe-4S] Cluster Assembly in Mitochondria and Its Impairment by Copper. J. Am. Chem. Soc..

[190] Sheftel A.D., Stehling O., Pierik A.J., Netz D.J.A., Kerscher S., Elsasser H.P., Wittig I., Balk J., Brandt U., Lill R. (2009). Human Ind1, an Iron-Sulfur Cluster Assembly Factor for Respiratory Complex I. Mol. Cell. Biol..

[191] Bych K., Kerscher S., Netz D.J.A., Pierik A.J., Zwicker K., Huynen M.A., Lill R., Brandt U., Balk J. (2008). The iron-sulphur protein Ind1 is required for effective complex I assembly. EMBO J..

[192] Bandyopadhyay S., Naik S.G., O′Carroll I.P., Huynh B.H., Dean D.R., Johnson M.K., Dos Santos P.C. (2008). A proposed role for the *Azotobacter vinelandii* NfuA protein as an intermediate iron-sulfur cluster carrier. J. Biol. Chem..

[193] Angelini S., Gerez C., Ollagnier-de Choudens S., Sanakis Y., Fontecave M., Barras F., Py B. (2008). NfuA, a new factor required for maturing Fe/S proteins in *Escherichia coli* under oxidative stress and iron starvation conditions. J. Biol. Chem..

[194] Tong W.H., Jameson G.N., Huynh B.H., Rouault T.A. (2003). Subcellular compartmentalization of human Nfu, an iron-sulfur cluster scaffold protein, and its ability to assemble a [4Fe-4S] cluster. Proc. Nat. Acad. Sci. USA.

[195] Ahting U., Mayr J.A., Vanlander A.V., Hardy S.A., Santra S., Makowski C., Alston C.L., Zimmermann F.A., Abela L., Plecko B. (2015). Clinical, biochemical, and genetic spectrum of seven patients with NFU1 deficiency. Front. Genet..

[196] Cai K., Liu G., Frederick R.O., Xiao R., Montelione G.T., Markley J.L. (2016). Structural/Functional Properties of Human NFU1, an Intermediate [4Fe-4S] Carrier in Human Mitochondrial Iron-Sulfur Cluster Biogenesis. Structure.

[197] Yabe T., Yamashita E., Kikuchi A., Morimoto K., Nakagawa A., Tsukihara T., Nakai M. (2008). Structural analysis of Arabidopsis CnfU protein: An iron-sulfur cluster biosynthetic scaffold in chloroplasts. J. Mol. Biol..

[198] Gao H., Subramanian S., Couturier J., Naik S.G., Kim S.K., Leustek T., Knaff D.B., Wu H.C., Vignols F., Huynh B.H. (2013). Arabidopsis thaliana Nfu2 accommodates [2Fe-2S] or [4Fe-4S] clusters and is competent for in vitro maturation of chloroplast [2Fe-2S] and [4Fe-4S] cluster-containing proteins. Biochemistry.

[199] McCarthy E.L., Booker S.J. (2017). Destruction and reformation of an iron-sulfur cluster during catalysis by lipoyl synthase. Science.

[200] Li H., Outten C.E. (2012). Monothiol CGFS glutaredoxins and BolA-like proteins: [2Fe-2S] Binding partners in iron homeostasis. Biochemistry.

[201] Willems P., Wanschers B.F.J., Esseling J., Szklarczyk R., Kudla U., Duarte I., Forkink M., Nooteboom M., Swarts H., Gloerich J. (2013). BOLA1 Is an Aerobic Protein That Prevents Mitochondrial Morphology Changes Induced by Glutathione Depletion. Antioxid. Redox Signal..

[202] Uzarska M.A., Nasta V., Weiler B.D., Spantgar F., Ciofi-Baffoni S., Saviello M.R., Gonnelli L., Muhlenhoff U., Banci L., Lill R. (2016). Mitochondrial Bol1 and Bol3 function as assembly factors for specific iron-sulfur proteins. Elife.

[203] Roret T., Tsan P., Couturier J., Zhang B., Johnson M.K., Rouhier N., Didierjean C. (2014). Structural and spectroscopic insights into BolA-glutaredoxin complexes. J. Biol. Chem..

[204] Kasai T., Inoue M., Koshiba S., Yabuki T., Aoki M., Nunokawa E., Seki E., Matsuda T., Matsuda N., Tomo Y. (2004). Solution structure of a BolA-like protein from Mus musculus. Protein Sci..

[205] Nasta V., Giachetti A., Ciofi-Baffoni S., Banci L. (2017). Structural insights into the molecular function of human [2Fe-2S] BOLA1-GRX5 and [2Fe-2S] BOLA3-GRX5 complexes. Biochim. Biophys. Acta.

[206] Muhlenhoff U., Molik S., Godoy J.R., Uzarska M.A., Richter N., Seubert A., Zhang Y., Stubbe J., Pierrel F., Herrero E. (2010). Cytosolic monothiol glutaredoxins function in intracellular iron sensing and trafficking via their bound iron-sulfur cluster. Cell Metab..

[207] Paul V.D., Lill R. (2015). Biogenesis of cytosolic and nuclear iron-sulfur proteins and their role in genome stability. Biochim. Biophys. Acta.

[208] Lill R., Srinivasan V., Muhlenhoff U. (2014). The role of mitochondria in cytosolic-nuclear iron-sulfur protein biogenesis and in cellular iron regulation. Curr. Opin. Microbiol..

[209] Sharma A.K., Pallesen L.J., Spang R.J., Walden W.E. (2010). Cytosolic iron-sulfur cluster assembly (CIA) system: Factors, mechanism, and relevance to cellular iron regulation. J. Biol. Chem..

[210] Pandey A., Pain J., Dziuba N., Pandey A.K., Dancis A., Lindahl P.A., Pain D. (2018). Mitochondria Export Sulfur Species Required for Cytosolic tRNA Thiolation. Cell Chem. Biol..

[211] Stehling O., Netz D.J., Niggemeyer B., Rosser R., Eisenstein R.S., Puccio H., Pierik A.J., Lill R. (2008). Human Nbp35 is essential for both cytosolic iron-sulfur protein assembly and iron homeostasis. Mol. Cell. Biol..

[212] Netz D.J., Pierik A.J., Stumpfig M., Muhlenhoff U., Lill R. (2007). The Cfd1-Nbp35 complex acts as a scaffold for iron-sulfur protein assembly in the yeast cytosol. Nat. Chem. Biol..

[213] Song D., Lee F.S. (2008). A role for IOP1 in mammalian cytosolic iron-sulfur protein biogenesis. J. Biol. Chem..

[214] Balk J., Netz D.J.A., Tepper K., Pierik A.J., Lill R. (2005). The essential WD40 protein Cia1 is involved in a late step of cytosolic and nuclear iron-sulfur protein assembly. Mol. Cell. Biol..

[215] Stehling O., Mascarenhas J., Vashisht A.A., Sheftel A.D., Niggemeyer B., Rosser R., Pierik A.J., Wohlschlegel J.A., Lill R. (2013). Human CIA2A-FAM96A and CIA2B-FAM96B integrate iron homeostasis and maturation of different subsets of cytosolic-nuclear iron-sulfur proteins. Cell Metab..

[216] Seki M., Takeda Y., Iwai K., Tanaka K. (2013). IOP1 Protein Is an External Component of the Human Cytosolic Iron-Sulfur Cluster Assembly (CIA) Machinery and Functions in the MMS19 Protein-dependent CIA Pathway. J. Biol. Chem..

[217] Stehling O., Vashisht A.A., Mascarenhas J., Jonsson Z.O., Sharma T., Netz D.J., Pierik A.J., Wohlschlegel J.A., Lill R. (2012). MMS19 assembles iron-sulfur proteins required for DNA metabolism and genomic integrity. Science.

[218] Ouyang B.J., Wang L., Wan S., Luo Y., Wang L., Lin J., Xia B. (2013). Solution structure of monomeric human FAM96A. J. Biomol. NMR.

[219] Chen K.E., Richards A.A., Ariffin J.K., Ross I.L., Sweet M.J., Kellie S., Kobe B., Martin J.L. (2012). The mammalian DUF59 protein Fam96a forms two distinct types of domain-swapped dimer. Acta Crystallogr. Sect. D Biol. Crystallogr..

[220] Maione V., Cantini F., Severi M., Banci L. (2018). Investigating the role of the human CIA2A-CIAO1 complex in the maturation of aconitase. Biochim. Biophys. Acta.

[221] Paul V.D., Muhlenhoff U., Stumpfig M., Seebacher J., Kugler K.G., Renicke C., Taxis C., Gavin A.C., Pierik A.J., Lill R. (2015). The deca-GX3 proteins Yae1-Lto1 function as adaptors recruiting the ABC protein Rli1 for iron-sulfur cluster insertion. Elife.

[222] Gari K., Leon Ortiz A.M., Borel V., Flynn H., Skehel J.M., Boulton S.J. (2012). MMS19 links cytoplasmic iron-sulfur cluster assembly to DNA metabolism. Science.

[223] Kim K.S., Maio N., Singh A., Rouault T.A. (2018). Cytosolic HSC20 integrates *de novo* iron-sulfur cluster biogenesis with the CIAO1-mediated transfer to recipients. Hum. Mol. Genet..

[224] Banci L., Bertini I., Calderone V., Ciofi-Baffoni S., Giachetti A., Jaiswal D., Mikolajczyk M., Piccioli M., Winkelmann J. (2013). Molecular view of an electron transfer process essential for iron-sulfur protein biogenesis. Proc. Natl. Acad. Sci. USA.

[225] Netz D.J., Stumpfig M., Dore C., Muhlenhoff U., Pierik A.J., Lill R. (2010). Tah18 transfers electrons to Dre2 in cytosolic iron-sulfur protein biogenesis. Nat. Chem. Biol..

[226] Frey A.G., Palenchar D.J., Wildemann J.D., Philpott C.C. (2016). A Glutaredoxin center dot BolA Complex Serves as an Iron-Sulfur Cluster Chaperone for the Cytosolic Cluster Assembly Machinery. J. Biol. Chem..

[227] Ojeda L., Keller G., Muhlenhoff U., Rutherford J.C., Lill R., Winge D.R. (2006). Role of glutaredoxin-3 and glutaredoxin-4 in the iron regulation of the Aft1 transcriptional activator in Saccharomyces cerevisiae. J. Biol. Chem..

[228] Banci L., Camponeschi F., Ciofi-Baffoni S., Muzzioli R. (2015). Elucidating the Molecular Function of Human BOLA2 in GRX3-Dependent Anamorsin Maturation Pathway. J. Am. Chem. Soc..

[229] Song D., Lee F.S. (2011). Mouse knock-out of IOP1 protein reveals its essential role in mammalian cytosolic iron-sulfur protein biogenesis. J. Biol. Chem..

[230] Banci L., Bertini I., Ciofi-Baffoni S., Boscaro F., Chatzi A., Mikolajczyk M., Tokatlidis K., Winkelmann J. (2011). Anamorsin Is a [2Fe-2S] Cluster-Containing Substrate of the Mia40-Dependent Mitochondrial Protein Trapping Machinery. Chem. Biol..

[231] Zhang Y., Yang C., Dancis A., Nakamaru-Ogiso E. (2017). EPR studies of wild type and mutant Dre2 identify essential [2Fe-2S] and [4Fe-4S] clusters and their cysteine ligands. J. Biochem..

[232] Banci L., Ciofi-Baffoni S., Mikolajczyk M., Winkelmann J., Bill E., Pandelia M.E. (2013). Human anamorsin binds [2Fe-2S] clusters with unique electronic properties. J. Biol. Inorg. Chem..

[233] Outten C.E., Albetel A.N. (2013). Iron sensing and regulation in Saccharomyces cerevisiae: Ironing out the mechanistic details. Curr. Opin. Microbiol..

[234] Li H., Mapolelo D.T., Randeniya S., Johnson M.K., Outten C.E. (2012). Human glutaredoxin 3 forms [2Fe-2S] -bridged complexes with human BolA2. Biochemistry.

[235] Banci L., Ciofi-Baffoni S., Gajda K., Muzzioli R., Peruzzini R., Winkelmann J. (2015). N-terminal domains mediate [2Fe-2S] cluster transfer from glutaredoxin-3 to anamorsin. Nat. Chem. Biol..

[236] Tamir S., Paddock M.L., Darash-Yahana-Baram M., Holt S.H., Sohn Y.S., Agranat L., Michaeli D., Stofleth J.T., Lipper C.H., Morcos F. (2015). Structure-function analysis of NEET proteins uncovers their role as key regulators of iron and ROS homeostasis in health and disease. Biochim. Biophys..

[237] Geldenhuys W.J., Leeper T.C., Carroll R.T. (2014). MitoNEET as a novel drug target for mitochondrial dysfunction. Drug Discov. Today.

[238] Bai F., Morcos F., Sohn Y.S., Darash-Yahana M., Rezende C.O., Lipper C.H., Paddock M.L., Song L.H., Luo Y.T., Holt S.H. (2015). The Fe-S cluster-containing NEET proteins mitoNEET and NAF-1 as chemotherapeutic targets in breast cancer. Proc. Nat. Acad. Sci. USA.

[239] Lin J., Zhou T., Ye K., Wang J. (2007). Crystal structure of human mitoNEET reveals distinct groups of iron sulfur proteins. Proc. Nat. Acad. Sci. USA.

[240] Ferecatu I., Goncalves S., Golinelli-Cohen M.P., Clemancey M., Martelli A., Riquier S., Guittet E., Latour J.M., Puccio H., Drapier J.C. (2014). The diabetes drug target MitoNEET governs a novel trafficking pathway to rebuild an Fe-S cluster into cytosolic aconitase/iron regulatory protein 1. J. Biol. Chem..

[241] Golinelli-Cohen M.P., Lescop E., Mons C., Goncalves S., Clemancey M., Santolini J., Guittet E., Blondin G., Latour J.M., Bouton C. (2016). Redox Control of the Human Iron-Sulfur Repair Protein MitoNEET Activity via Its Iron-Sulfur Cluster. J. Biol. Chem..

[242] Andreini C., Banci L., Rosato A. (2016). Exploiting Bacterial Operons to Illuminate Human Iron-Sulfur Proteins. J. Proteome Res..

[243] Camponeschi F., Ciofi-Baffoni S., Banci L. (2017). Anamorsin/Ndor1 Complex Reduces [2Fe-2S]-MitoNEET via a Transient Protein-Protein Interaction. J. Am. Chem. Soc..

[244] Luchinat E., Banci L. (2018). In-Cell NMR in Human Cells: Direct Protein Expression Allows Structural Studies of Protein Folding and Maturation. Acc. Chem. Res..

[245] Luchinat E., Banci L. (2016). A Unique Tool for Cellular Structural Biology: In-cell NMR. J. Biol. Chem..

[246] Freedberg D.I., Selenko P. (2014). Live cell NMR. Annu. Rev. Biophys..

[247] Maldonado A.Y., Burz D.S., Shekhtman A. (2011). In-cell NMR spectroscopy. Prog. Nucl. Magn. Reson. Spectrosc..

[248] Sakakibara D., Sasaki A., Ikeya T., Hamatsu J., Hanashima T., Mishima M., Yoshimasu M., Hayashi N., Mikawa T., Walchli M. (2009). Protein structure determination in living cells by in-cell NMR spectroscopy. Nature.

[249] Inomata K., Ohno A., Tochio H., Isogai S., Tenno T., Nakase I., Takeuchi T., Futaki S., Ito Y., Hiroaki H. (2009). High-resolution multi-dimensional NMR spectroscopy of proteins in human cells. Nature.

